# CRISPR-typing PCR (ctPCR), a new Cas9-based DNA detection method

**DOI:** 10.1038/s41598-018-32329-x

**Published:** 2018-09-20

**Authors:** Qiao Wang, Beibei Zhang, Xinhui Xu, Feifei Long, Jinke Wang

**Affiliations:** 1 State Key Laboratory of Bioelectronics, Southeast University, Nanjing, 210096 China; 2 Nanjing Foreign Language School, Nanjing, 210096 China

## Abstract

This study develops a new method for detecting and typing target DNA based on Cas9 nuclease, which was named as ctPCR, representing Cas9-sgRNA- or CRISPR-typing PCR. The technique can detect and type target DNA easily, rapidly, specifically, and sensitively. This technique detects target DNA in three steps: (1) amplifying target DNA with PCR by using a pair of universal primers (PCR1); (2) treating PCR1 products with a process referred to as CAT, representing Cas9 cutting, A tailing and T adaptor ligation; (3) amplifying the CAT-treated DNA with PCR by using a pair of general-specific primers (gs-primers) (PCR2). This method was verified by detecting HPV16 and HPV18 L1 gene in 13 different high-risk human papillomavirus (HPV) subtypes. This method was also verified by detecting the L1 and E6-E7 genes of two high-risk HPVs (HPV16 and 18) in cervical carcinoma cells and many clinical samples. In this method, PCR1 was performed to determine if the detected DNA sample contained the target DNA (such as virus infection), while PCR2 was performed to discriminate which genotypic target DNA was present in the detected DNA sample (such as virus subtypes). Based on these proof-of-concept experiments, this study provides a new CRISPR/Cas9-based DNA detection and typing method.

## Introduction

DNA detection and genotyping are always of importance to the basic researches and various detecting and diagnostic applications. Therefore, the DNA detection and genotyping techniques have been attracting increasing attention, which promotes the advance of techniques. Summarily, there are mainly three classes of widely used DNA detection and genotyping techniques. The first is various techniques based on polymerase chain reaction (PCR). PCR has been the most popular DNA detection and genotyping technique. PCR-based DNA detection and genotyping mainly relies on the design of highly specific PCR primers and multiple PCR amplification. PCR detection can be realized by the traditional PCR (tPCR), the quantitative PCR (qPCR), and the recently developed digital PCR^1^. Q-PCR has been highly popularized in almost all basic research and diagnostic labs due to its obvious advantages such as real time and high sensitivity. Now, the digital PCR is becoming more and more accurate, which can be used as clinical detection tool for its huge potential and advantages^2,3^. However, the PCR techniques are still limited by the difficulty of multiple amplification and the design of highly specific primers that can discriminate highly-related genotypes. The second is various DNA hybridization techniques such as DNA microarray^4,5^. However, in consideration of its costly equipment, complicate detection process and unavoidable nonspecific hybridization, DNA microarray technique is unable to become a routine DNA detection and genotyping tool. The third is various DNA sequencing techniques^6,7^. Especially, with the advent of next-generation sequencing (NGS) techniques^8,9^, the NGS platforms such as Illumina NovaSeq become available DNA sequencing tools increasingly. Nevertheless, considering their costly equipment and chemical reagents currently, they are still prohibitive to the routine research, detection and diagnosis. Comparatively, PCR is still a most convenient, cost-effective, and ready platform for DNA detection and genotyping, especially if its primer designing limitation is overcome.

The clustered regularly interspaced short palindromic repeat (CRISPR) is a new gene editing tool, in which the CRISPR/Cas9 system is most widely used to edit genes due to its simplicity and efficiency^10^. Additionally, the dead Cas9 (dCas9) has been widely used to regulate gene expression by fusing with a gene transcriptional activation domain (AD) or inhibition domain (ID)^11^. Despite its wide application in gene editing and regulation, the CRISPR/Cas9 system was not fully exploited to nucleic acid detection. However, due to the sequence-specific DNA cutting capability^12^, the Cas9 and its cognate single guide RNA (sgRNA) have great potential in DNA detection and typing. For example, the Cas9-sgRNA was used to eliminate the wild-type genotypes in blood cell free DNA (cfDNA), so that the disease-associated mutant genotypes can be detected by PCR more easily^13^. The CRISPR/Cas9 system was recently used to type American and African Zika viruses in a RNA *in vitro* transcription-based Zika virus detection technique^14^. Given the high specificity of CRISPR-based tools, Cas9-sgRNA can discriminate viral strains at single-base resolution^14^, which is beneficial for detecting and typing the orthologous and homologous bacteria and viruses even at single nucleotide polymorphism level. Other CRISPR systems, such as Cas13a/C2c2, have been recently used to develop techniques for detecting Zika virus, which has an ultrahigh sensitivity (viral particles down to 2 aM)^15^. These studies indicate that the CRISPR systems have a great potential and advantages to be exploited to develop nucleic acid detection techniques. However, in the currently reported Cas9-based nucleic detection method^14^, the Cas9-sgRNA system was used to type RNAs by cutting DNA reversely transcribed from the detected RNAs. The Cas9-sgRNA system therefore has not been exploited to directly detect and type various DNA targets, which is the main object of routine nucleic acid detection.

To explore the potential of Cas9-based DNA detection, we have recently developed a Cas9-based reverse PCR technique, in which the Cas9-cut target DNA was cyclized and detected by a reverse PCR amplification^16^. Despite the technique still limited by the difficulty of designing specific reverse PCR primers, the great potential of CRISPR/Cas9 application in DNA detection was preliminarily demonstrated. To further explore the applications of Cas9-sgRNA, this study developed a new Cas9-based DNA detection technique, which was named as ctPCR, representing the Cas9-sgRNA- or CRISPR-typing PCR. In the ctPCR detection, the target DNA was firstly amplified by a first-round PCR (PCR1) with a pair of universal primers. The PCR1 products were then successively digested by Cas9 in complex with a pair of sgRNAs, tailed by an adenine (A), and ligated by a T adaptor. Finally, the treated PCR1 products were amplified by a second-round PCR (PCR2) using a pair of general-specific primers (gs-primers). PCR1 was used to find whether the DNA sample contained the target DNA (such as virus infection), while PCR2 was used to discriminate which genotype of DNA presents in the DNA sample (such as virus subtype). This study demonstrated that ctPCR could detect the L1 genes of HPV16 and HPV18 from 11 different high-risk human papillomavirus (HPV) subtypes. This study also indicated that ctPCR could detect two high-risk HPVs (HPV16 and HPV18) in the human cervical carcinoma cells (HeLa and SiHa) and clinical samples by detecting both L1 and E6/E7 genes. By performing these proof-of-principle detections, this study developed a new CRISPR-based PCR technique for detecting and typing DNA. This technique takes advantages of PCR and CRISPR. This technique realized rapid DNA detection and typing without depending on hybridization and sequencing.

## Experimental Section

### *In vivo* cleavage of HPV16 and 18 L1 genes with Cas9-sgRNA

The sgRNA and Cas9 expression plasmids were constructed as described in detail in the Supplemental Information. For the *in vivo* cutting of HPV L1 genes with Cas9-sgRNA, *E*. *coli* DH5α was firstly transformed with the plasmids cloned with HPV L1 gene and an ampicillin resistance gene (Amp^R^) under the control of Amp^R^ promoter. The transformed *E*. *coli* was selected on ampicillin agars and positive *E*. *coli* clones were confirmed by a clone PCR. The positive *E*. *coli* was then transformed with pCas9-sgRNA expressing various sgRNAs. The transformed *E*. *coli* was cultivated on agars with ampicillin plus chloromycetin overnight and imaged.

### Cleavage of HPV L1 genes cloned in plasmid with Cas9-sgRNA

SgRNAs were prepared by *in vitro* transcription as described in detail in the Supplemental Information. The recombinant Cas9 protein was purchased from New England Biolabs (NEB). The Cas9 digestion reaction (30 μL) consisted of 1× Cas9 Nuclease Reaction Buffer, 30 nM Cas9 Nuclease (NEB), 30 nM sgRNA a (16–1274 or 18–1490; Table 1) and 30 nM sgRNA b (16–950 or 18–1274; Table 1) was firstly incubated at 25 °C for 10 min (this process was referred to as pre-assemble hereafter). Two hundred ng of substrate DNA (L1 plasmid DNA linearized by AatII) mixed with above solution was incubated at 37 °C for 5 min. The reaction was mixed with 10× SDS-containing loading buffer (Takara) and run with 1.0% agarose gel.

**Table 1:** The target sequences of sgRNA (plus PAM) used for *in vivo* Cas9-sgRNA cutting.

Name	Sequence (5′ to 3′)	Name	Sequence (5′ to 3′)
6–146	AGTTCTAGACTTCTTGCAGTGGG	16–950	ACTGTGTTTATTAACTCTAATGG
11–146	AGTTCTAGACTCCTTGCTGTGGG	18–950	ACTGTGTTTTTAAGTTCTAAGGG
6–434	TAACCCTGGACAGGATAACAGGG	16–1274	AAACCAAACTTATTGGGGTCGGG
11–434	TAATCCTGGTCAGGATAATAGGG	18–1274	AAACCAAATTTATTTGGGTCAGG
6–978	CATAACAATGGTATTTGTTGGGG	16–1490	AAGTAGACAGTGGCCTCACTAGG
11–978	CATAACAATGGTATTTGCTGGGG	18–1490	AGATATACGGTATTGTCACTAGG

### Detection of HPV16 and 18 L1 genes cloned in plasmid with ctPCR

For preparing T adaptor, the oligo oJW102 and oJW103 (Table 2) were dissolved in the Tris-HCl/EDTA/NaCl (TEN) buffer and mixed in the same molar. The mixture was heated at 95 °C for 5 min, and slowly cooled to room temperature. Plasmids cloned with the L1 genes of various HPV subtypes (200 ng) were cut by the Cas9 proteins in complex with a pair of sgRNAs targeting to HPV16 and HPV18 L1 genes. The plasmid (200 ng) was mixed with the pre-assembled Cas9-sgRNA complex that contained 1× Cas9 nuclease reaction buffer, 30 nM Cas9 nuclease, 30 nM sgRNA a (16–1274 or 18–1490; Table 1), and 30 nM sgRNA b (16–950 or 18–1274; Table 1) and incubated at 37 °C for 5 min. The digestion reaction (5 μL) was mixed with 5 μL premix Taq (Takara) and incubated at 72 °C for 5 min. The A tailing reaction (10 μL) was mixed with 1× T4 Ligase Buffer, 830 nM T adaptor and 5 U T4 DNA Ligase and incubated at 22 °C for 5 min. The process of Cas9 cutting, A tailing and T adaptor ligation was concisely referred to as CAT hereafter. At last, the CAT-treated DNAs were amplified with tPCR by using a general primer (oJW102) annealing to T adaptor or a pair of general-specific primers (gs-primers) specific to the Cas9-digested HPV16 and HPV18 L1 genes. The tPCR reaction contained 10 μL SYBR Green (Bioer), 500 nM of a general primer (oJW102; Table 2) or 500 nM of each gs-primers specific to the L1 and E6-E7 genes of HPV16 and 18 (Table 2). The PCR program was as follows: 95 °C 2 min, 30 cycles of 95 °C 15 s, 60 °C 30 s and 72 °C 60 s, and 72 °C 5 min. The reaction was run with 1.5% agarose gel.

**Table 2:** Oligonucleotides used as T adaptor and PCR primers in this study.

Name	Sequence (5′ to 3′) (*)	Name	Sequence (5′ to 3′) (*)
oJW102	GCGGTGACCCGGGAGATCTGAATTCT	oJW103	pGAATTCAGATC
L1-MY09	CGTCCMARRGGAWACTGATC	L1-MY11	GCMCAGGGWCATAAYAATGG
E67-6F	AAGGGMGTAACCGAAAWCGGT	E67-7R	GTACCTKCWGGATCAGCCAT
16L1F	GCGGTGACCCGGGAGATCTGAATTCT*GGG*	16L1R	GCGGTGACCCGGGAGATCTGAATTCT*TGG*
18L1F	GCGGTGACCCGGGAGATCTGAATTCT*CCT*	18L1R	GCGGTGACCCGGGAGATCTGAATTCT*GAC*
16E67F	GCGGTGACCCGGGAGATCTGAATTCT*GGT*	16E67R	GCGGTGACCCGGGAGATCTGAATTCT*ATT*
18E67F	GCGGTGACCCGGGAGATCTGAATTCT*GAC*	18E67R	GCGGTGACCCGGGAGATCTGAATTCT*AGT*

*The italic bases are the three specific nucleotides in the sg-primers.

### Detection of HPV DNAs in human cervical carcinoma cells with ctPCR

For the tPCR detection, tPCR1 amplification of L1 or E6-E7 genes was carried out in a 20-μL tPCR reaction containing 10 μL premix primeSTAR Taq (Takara), 500 nM MY09 or E67-6F (Table 2), 500 nM MY11 or E67-7R (Table 2), various amounts of gDNA (see figures) of three human cervical carcinoma cells (SiHa, HeLa and C-33a). The PCR program was as follows: 95 °C 2 min, 35 cycles of 95 °C 15 s, 60 °C 30 s and 72 °C 60 s, and 72 °C 5 min. The PCR products were run with agarose gel. The PCR1 products (5 μL) were treated with CAT. Finally the CAT-treated PCR1 products (1 μL) were amplified in a 20-μL tPCR2 reaction containing 10 μL SYBR Green (Bioer), 500 nM of each gs-primers specific to the L1 and E6-E7 genes of HPV16 and 18 (Table 2). The PCR program was as follows: 95 °C 2 min, 30 cycles of 95 °C 15 s, 60 °C 30 s and 72 °C 60 s, and 72 °C 5 min. PCR program was run on a tPCR machine, 9700 (ABI). The PCR2 products were run with 1.5% agarose gel.

For the qPCR detection, qPCR1 amplification of L1 or E6-E7 genes was carried out in a 20-μL qPCR reaction containing 10 μL 2× Sybr Green Master Mix (Yeasen) and other reagents same as in tPCR1. The qPCR program was as follows: 95 °C 10 min, 40 cycles of 95 °C 15 s, 60 °C 30 s and 72 °C 1 min. The qPCR1 products (2 μL) were treated with CAT. Finally the CAT-treated qPCR1 products (1 μL) were amplified in a 20-μL qPCR2 reaction containing 10 μL 2× Sybr Green Master Mix (Yeasen) and other reagents same as in tPCR2. The PCR program was same as qPCR1. PCR program was run on a real-time PCR machine, StepOne Plus (ABI). The qPCR1 and qPCR2 products were also sometimes run with 1.5% agarose gel for further checking specificity in addition to melting curve.

### Detection of HPV DNAs in the clinical samples with ctPCR

DNA was purified from the clinical samples (cervical mucus exfoliated cells) by phenol/chloroform extraction and precipitated with ethanol. The purified DNA was dissolved in ddH_2_O and quantified by spectrometry. The samples had already detected with the HC2 (Diogenes) test by the Jinling Hospital, Nanjing University School of Medicine. The HPV infection of these samples was thus known. The ctPCR detection of these samples was used to verify its feasibility and reliability to detection HPV clinical samples by repeating the results of HC2 test. For the qPCR detection of eight clinical samples (number 1–8), qPCR1 amplification of L1 or E6-E7 genes was carried out in a 20-μL qPCR reaction as described above. One hundred ng of DNA was used as template of qPCR1. The qPCR1 products (2 μL) were treated with CAT. Finally, the CAT-treated qPCR1 products (1 μL) were amplified in a 20-μL qPCR2 reaction as described above. PCR program was run on a real-time PCR machine, StepOne Plus (ABI).

To further demonstrate the capability and specificity of ctPCR to detect clinical samples, a second batch of clinical samples was detected by ctPCR. This batch of samples was detected in a double-blind format. These samples were independently detected by the Jinling Hospital with commercialized HPV detection kits, in which the HC2 (Diogenes) was used to determine if a sample was infected by high-risk HPVs and a PCR-Reverse dot blot (PCR-RDB) (Yaneng Bio) was used to determine genotypes. These samples were then numbered as Sample 1–10 and blindly detected again in our lab by ctPCR. When detection was finished, the ctPCR detection results were compared with those obtained by the Jinling Hospital. The procedures of DNA purification and ctPCR detection of these samples were completely same as the detection of the first batch of samples. The qPCR1 detection used the MY09 and MY11 primers.

### Detection sensitivity of qPCR-based ctPCR

In order to prepare a standard molecule, a HPV18 L1 fragment was amplified with the MY09 and MY11 primers by using the plasmid contained the HPV18 L1 gene as template. The PCR amplification was performed in a 50-μL reaction containing 80 ng of plasmid DNA (pHPV18), 1× premix primeSTAR Taq (Takara), 200 nM MY09, and 200 nM MY11. The PCR program was as follows: 95 °C 2 min, 35 cycles of 95 °C 15 s, 58 °C 30 s and 72 °C 60 s, and 72 °C 5 min. The PCR products were run with agarose gel. The unique band of MY09/11-amplified HPV18 L1 fragment (452 bp) was recovered from gel and purified with the AxyPrep DNA Gel Extraction kit. The purified DNA was quantified with NanoDrop 2000. The concentration of DNA was 40 ng/μl. The partial raw DNA solution (40 ng/μl) was diluted to 0.4 ng/μl, which was then used to prepare a series of dilutions of 10 fold. Totally seven dilutions were prepared with the highest concentration of 0.4 ng/μl. These dilutions were then detected with qPCR1 by using MY09 and MY11 primers. The PCR amplification was performed in a 20-μL reaction containing 1 μl variant dilutions, 1× Sybr Green Master Mix (Yeasen), 500 nM MY09, and 500 nM MY11. The PCR program was as follows: 95 °C 2 min, 40 cycles of 95 °C 15 s and 58 °C 60 s. According to the ng quality and the molecular weight, the copy number per μl was calculated. According to the Ct values and DNA copies, a standard curve with R^2^ over 0.99 was built. Then 5 μl qPCR1 reaction of the sixth dilution was treated with CAT. The CAT product was diluted in 10 fold and a series of dilutions were prepared. These dilutions were then detected with qPCR2 by using gs-18L1-F and gs-18L1-R primers. The PCR amplification was performed in a 20-μL reaction containing 1 μl variant dilutions, 1× Sybr Green Master Mix (Yeasen), 500 nM gs-18L1-F, and 500 nM gs-18L1-R. The PCR program was as follows: 95 °C 2 min, 40 cycles of 95 °C 15 s and 60 °C 60 s.

## Results

### Cleavage of HPV 16 and 18 L1 genes with Cas9-sgRNA

To preliminarily explore if the Cas9-sgRNA system could be used to specifically discriminate subtypes of HPVs, we firstly performed an *in vivo* cutting assay. In this assay, *E*. *coli* DH5α was firstly transformed with a HPV L1 plasmid that has ampicillin resistance. The positive cells were then transformed with a Cas9-sgRNA-expressing plasmid that has Chloromycetin resistance. After overnight cultivation, agar plates were imaged. We found that the *E*. *coli* with a HPV L1 plasmid was always specifically killed by Cas9 nuclease guided by sgRNA specific to the HPV L1 gene (Fig. S1). These results revealed that the designed sgRNA could specifically target to its target and the Cas9-sgRNA could be used to type HPV subtypes.

### Cleavage of HPV L1 genes cloned in plasmid with Cas9-sgRNA

**Figure 1:**
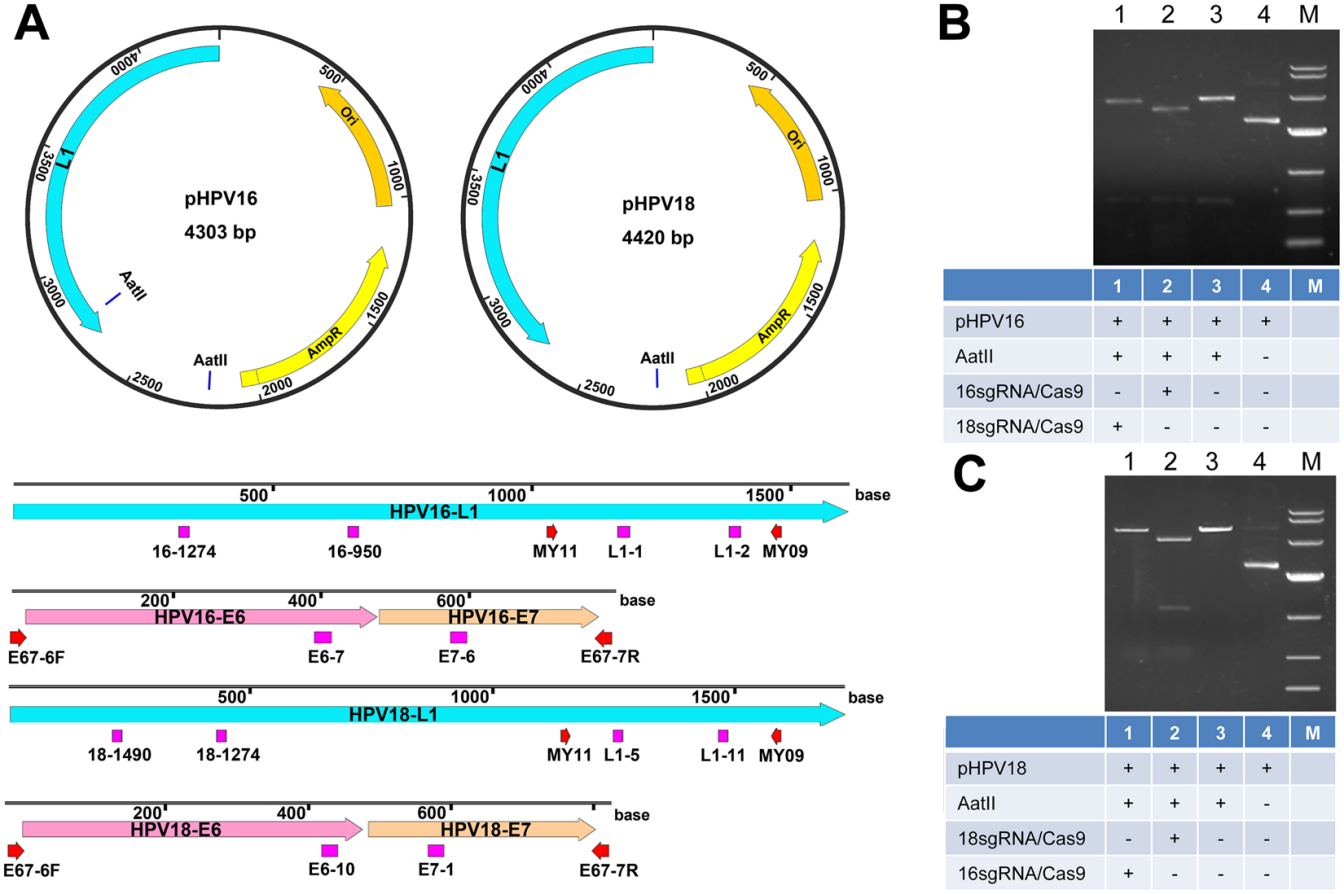
Cas9-sgRNA cleavage of HPV16 and HPV18 L1 genes. (**A**) HPV L1 plasmids and locations of sgRNA targets and universal PCR primers in the L1 and E6-E7 genes of HPV16 and HPV18. (**B**) Cas9-sgRNA cleavage of HPV16 L1 gene using sgRNA 16–1274 and 16–950. (**C**) Cas9-sgRNA cleavage of HPV18 L1 gene using sgRNA 18–1490 and 18–1274. The Cas9 protein was in complex with the sgRNAs specific to HPV16 or 18 L1 genes and used to cut the linearized HPV16 or HPV18 L1 plasmids (**A**). The DNAs were run with agarose gel.

With the high-efficiency *in vivo* cutting of HPV L1 genes by Cas9-sgRNA, we thought if the kind of specific DNA cutting by the sgRNA-guided Cas9 could be used to detect and type DNA *in vitro*. We thus performed the *in vitro* cutting assays. We firstly linearized the plasmids cloned with the HPV16 and HPV18 L1 genes with a restriction endonuclease, AatII, which produced linear DNA fragments ended with a 4-base 3′ overhang. We cut the linearized HPV16 and HPV18 L1 plasmid DNAs with Cas9 nuclease in complex with the sgRNAs specific to HPV16 and HPV18 L1 genes (Table 1). The results indicated that the HPV16 and HPV18 L1 genes could be specifically targeted by their corresponding sgRNA and cut by the guided Cas9 nuclease (Fig. 1). This means that the *in vitro* specific DNA cutting by Cas9-sgRNA could be used to detect and type DNA.

### Detection of HPV16 and 18 L1 genes with ctPCR

**Figure 2:**
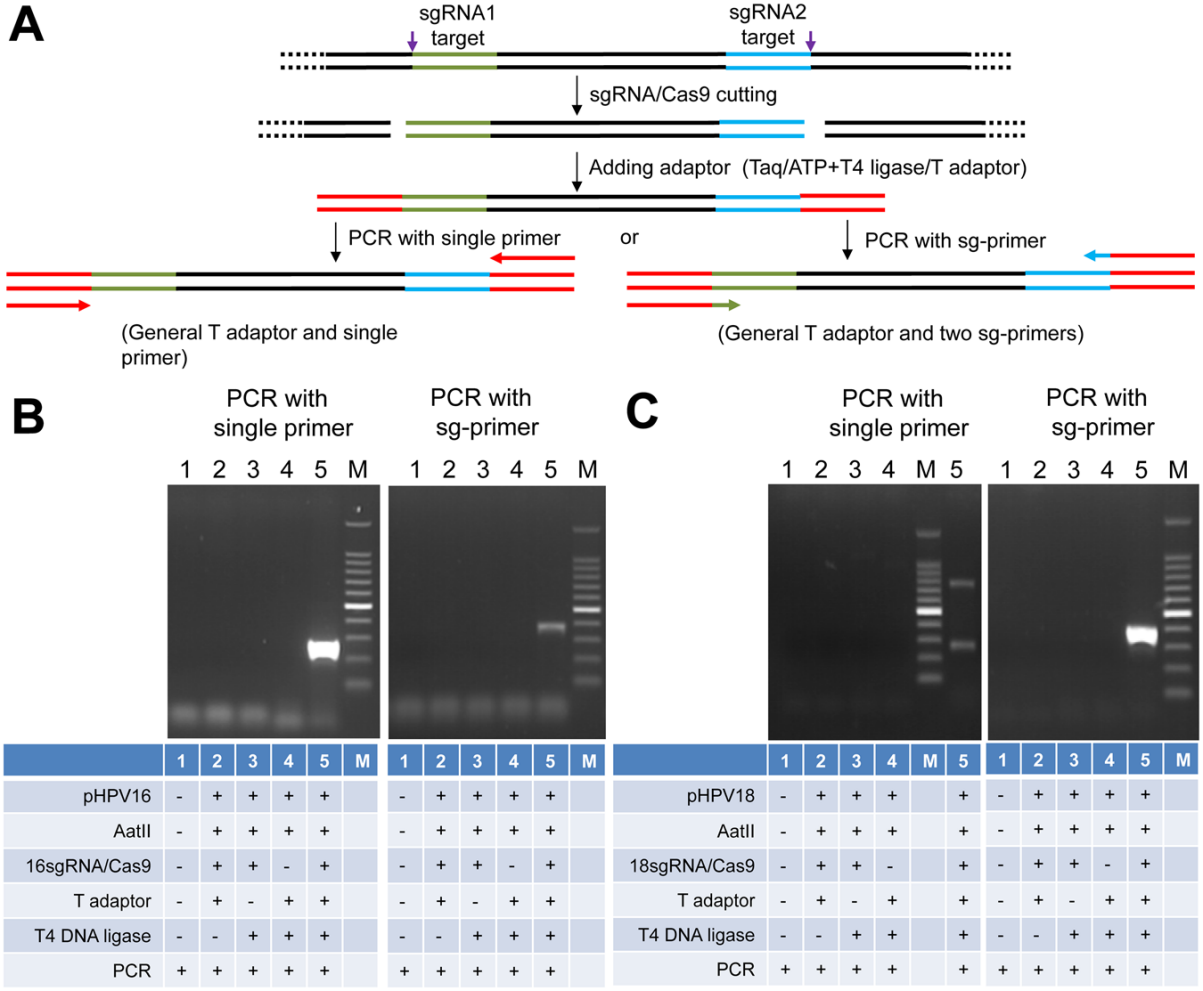
Detection of HPV L1 gene with ctPCR by using different primers. (**A**) A schematic of the experimental procedures for detecting and typing HPV DNA with ctPCR by using different primers. (**B**) Detection of HPV16 L1 gene by ctPCR with different primers. (**C**) Detection of HPV18 L1 gene by ctPCR with different primers. The final PCR products were run with agarose gel. Single primer, a general primer complementary to the constant T adaptor used in ctPCR detection; Sg-primer, a pair of primers complementary to constant T adaptor and 3 nucleotides at the end of Cas9-cut product (named as general-specific primers, gs-primers).

To detect and type DNA with Cas9-sgRNA, we designed a CRISPR-typing PCR (ctPCR) method for realizing this end. In this method, a target DNA was firstly cut with a pair of sgRNAs specific to the interested target DNA. The Cas9-sgRNA-cut DNA was then tailed with an adenine (A) and ligated with a T adaptor. The process of Cas9 cutting, A tailing and T adaptor ligation was referred to as CAT hereafter. Finally, the CAT-treated DNA was amplified with PCR by using a constant primer annealing to T adaptor (single primer) (Fig. 2A). We used the method detected HPV16 and HPV18. The results revealed that the expected HPV16 target DNA was successfully and specifically detected by the method (Fig. 2B), while there was an additional nonspecific DNA fragment when detecting HPV18 by the method (Fig. 2C). In order to improve the detection specificity, we added three specific nucleotides at the 3′ end of the constant primer annealing to T adaptor. These specific nucleotides are specific to the three nucleotides at the ends of the target DNA fragment cut out by Cas9-sgRNA (Fig. 2A). We named this kind of primer as general-specific primer (gs-primer). We prepared a pair of gs-primers for HPV16 and HPV18 according to the cutting sites of HPV16 and HPV18 sgRNAs (Table 1). We then amplified the CAT-treated DNA with a pair of gs-primers. As a result, we found that the target HPV16 and HPV18 DNAs were specifically detected by the improved ctPCR method (Fig. 2B and C).

### Sensitivity of ctPCR detection of HPV16 and HPV18 L1 gene

We next investigated the detection sensitivity of L1 gene with ctPCR. Different amounts of HPV16 and HPV18 L1 gene plasmids were respectively cut with the Cas9 nuclease in complex with pairs of sgRNAs targeting to the HPV16 and HPV18 L1 genes. The cut DNA was tailed with A and ligated with T adaptor. Then the CAT-treated DNA was amplified by PCR with the corresponding pairs of gs-primers and the PCR products were detected with agarose gel electrophoresis. The results revealed that ctPCR showed high amplification efficiency and sensitivity. It was found that as little as 5 ng of CAT-treated HPV18 L1 plasmid DNA could be detected by the tPCR-based ctPCR (Fig. S2). In addition, when the CAT-treated HPV18 L1 plasmid DNA was amplified by qPCR, as little as 10000-fold diluted cat-treated HPV18 L1 plasmid DNA could be detected. These data suggested that the CAT treatment had high efficiency.

### Detection of HPV 16 or 18 L1 genes in 13 HPV subtypes with ctPCR

In order to further verify the specificity of ctPCR, we digested the L1 genes of 12 HPV subtypes with the Cas9 protein in complex with the sgRNAs of HPV16 or HPV18. The digested DNAs were then tailed with A and ligated with T adaptor. The CAT-treated DNAs were amplified with tPCR by using gs-primers. Finally, the tPCR products were run with agarose gel. The results revealed that the L1 genes of HPV16 and HPV18 were specifically detected by ctPCR from other 11 HPV subtypes (Fig. S3). Two highest risk HPV subtypes, HPV16 and HPV18, were discriminated from other 10 high-risk HPV subtypes.

### Detection of HPV genes in the cervical carcinoma cells with ctPCR

**Figure 3:**
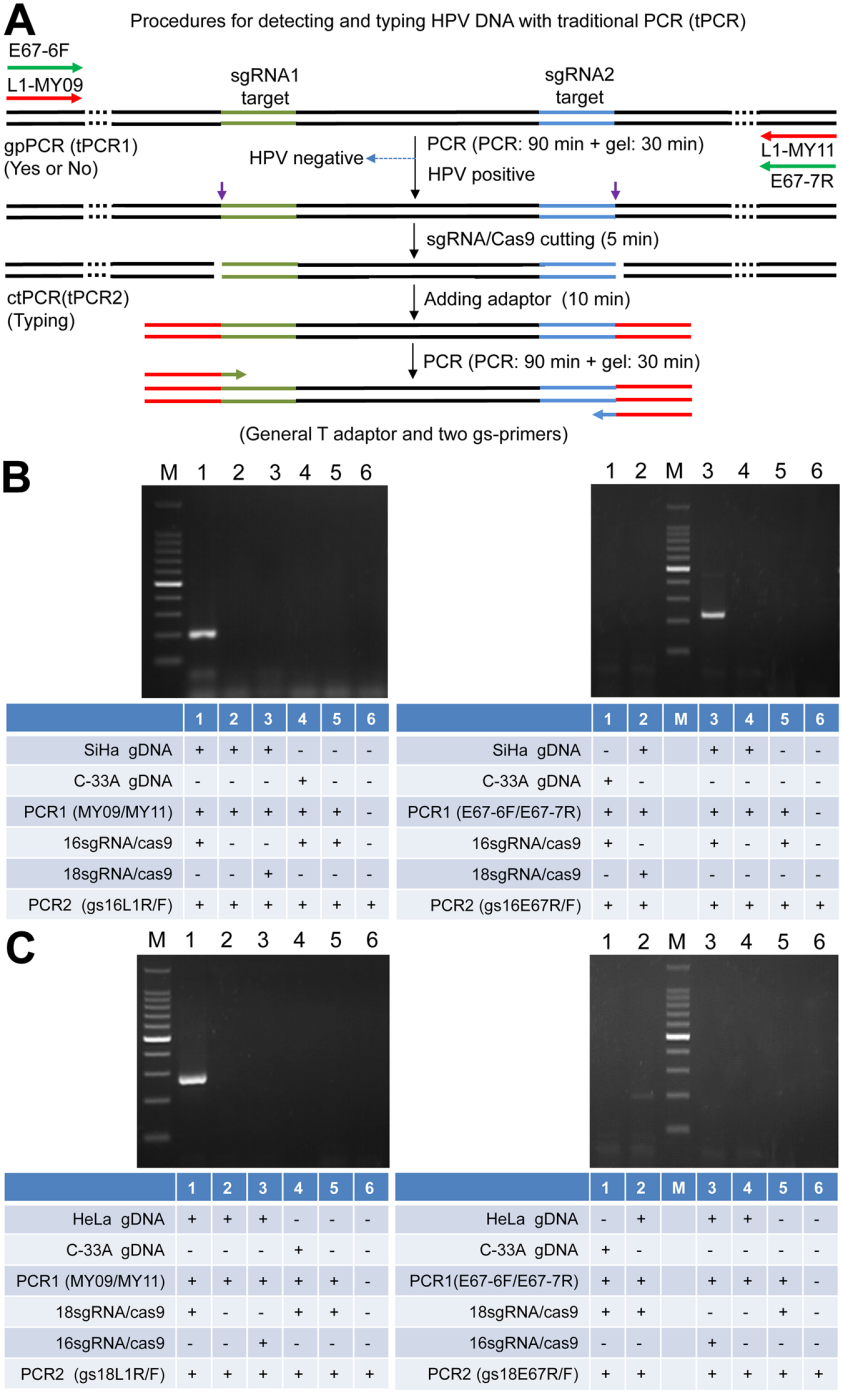
Detection of HPV16 and HPV 18 genes in cervical carcinoma cells with tPCR-based ctPCR. (**A**) Schematic show of procedures for detecting and typing HPV DNA with tPCR-based ctPCR. (**B**) Detecting the HPV16 L1 and E6-E7 genes in the SiHa gDNA (200 ng) with ctPCR. (**C**) Detecting the HPV18 L1 and E6-E7 genes in the HeLa gDNA (200 ng) with ctPCR. The C-33a gDNA (200 ng) was detected as a negative control and a mimic detection of no DNA was used as a blank control. The final ctPCR products were run with agarose gel.

Despite the L1 genes could be detected by ctPCR, the L1 genes together with its host plasmid is a relative simple DNA sample to ctPCR detection. However, the HPV clinical detection uses the complex genomic DNAs (gDNAs) of cells. To check if the ctPCR technique could be used to detect HPV in gDNAs, we next tried to detect the L1 and E6-E7 genes of HPVs in the gDNAs of human cervical carcinoma cells according to a two-round PCR strategy (Fig. 3A). To this end, we prepared the gDNAs from three human cervical carcinoma cell lines, HeLa, SiHa and C-33a. We then amplified the L1 gene by using a pair of universal primers, MY09 and MY11, which was previously designed to amplified the L1 genes of various HPV subtypes. As a result, the L1 gene was successfully amplified from the HeLa and SiHa gDNAs but not amplified from the C-33a gDNA (Fig. S4). We also designed a pair of universal primers, E67-6F and E67-7R, for amplifying the E6-E7 genes of various HPV subtypes. The results revealed that the E6-E7 genes could be amplified from the HeLa and SiHa gDNAs but not amplified from the C-33a gDNA (Fig. S4). We called this first-round PCR as tPCR1 (Fig. 3A). We treated the L1 and E6-E7 tPCR1 products with CAT. The CAT-treated tPCR1 products were detected with a tPCR2 by using pairs of gs-primers specific to the L1 and E6-E7 genes of HPV16 and HPV18 (Fig. 3A). Although no L1 and E6-E7 genes were detected from the C-33a gDNA by tPCR1, we still treated the tPCR1 products of the C-33a gDNA with CAT and amplified with tPCR2. However, no L1 and E6-E7 genes of HPV16 and 18 were detected (Fig. 3B and C). These results are in agreement with the previous reports that HeLa and SiHa are HPV18- and HPV16-positive cells^17,18^, respectively, and C-33a is a HPV-negative cell^19^.

**Figure 4:**
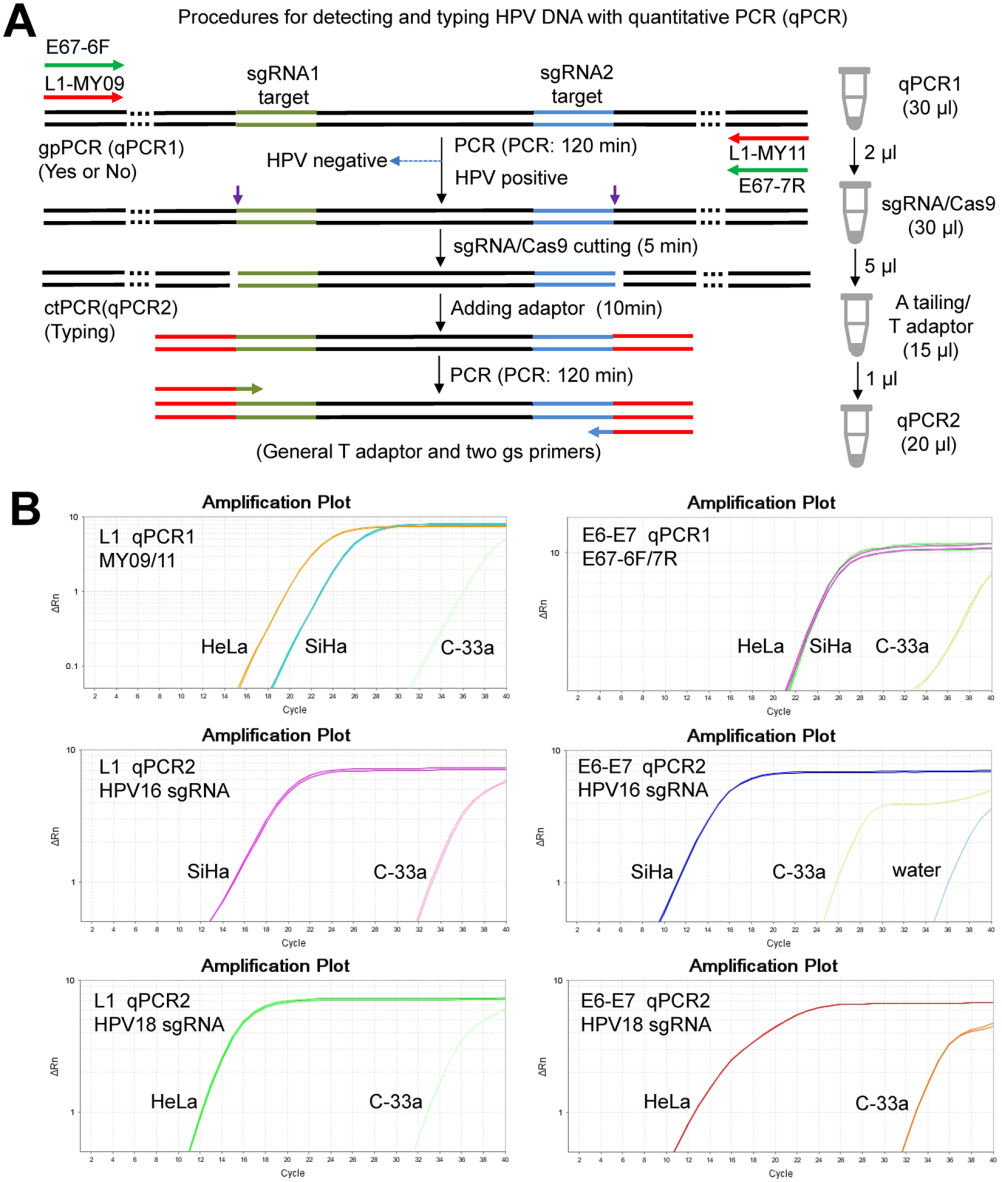
Detection of HPV16 and HPV 18 genes in cervical carcinoma cells with qPCR-based ctPCR. (**A**) Schematic show of procedures for detecting and typing HPV DNA with qPCR-based ctPCR. The reaction volumes of each step and the solutions used to the next step were shown (right). (**B**) Detection of the HPV16 L1 and E6-E7 genes in the three human cervical carcinoma cell lines, HeLa, SiHa and C-33a. Q-PCR1 used 200 ng gDNA of each cell line as template.

Despite the L1 and E6-E7 genes could be detected by ctPCR from the gDNAs of human cervical carcinoma cells, such tPCR-based ctPCR detection is unfavorable to its clinical application due to cumbersome gel electrophoresis. We therefore investigated if the ctPCR detection could be realized with a similar two-round qPCR (Fig. 4A). As expected, both L1 and E6-E7 genes were easily amplified by qPCR1 from gDNAs of HeLa and SiHa cells (Fig. 4B and C). We next treated the qPCR1 products with CAT and amplified with qPCR2 by using gs-primers. As expected, the HPV16 and HPV18 L1 and E6-E7 genes were successfully detected in the gDNA of SiHa and HeLa cells, respectively (Fig. 4B and C).

In order to check the detection specificity of ctPCR, we used as many as 200 ng gDNA as PCR1 template to detected HPV L1 and E6-E7 genes in the tPCR and qPCR detection. We next checked the detection sensitivity of both qPCR1 and qPCR2. To this end, we amplified the HPV18 L1 gene from various amount of HeLa gDNAs with qPCR1. The results indicated that the HPV18 L1 gene could be amplified from as little as 5 pg gDNA (Fig. S5A). In addition, even the CAT-treated qPCR1 product of 5 pg gDNA template was diluted 1000 fold (10^−3^), qPCR2 could amplify the HPV18 L1 gene using 1 μL of the diluted solution (Fig. S5B). These results indicated that both qPCR1 and qPCR2 had high sensitivity, suggesting that the qPCR-based ctPCR is favorable to clinical application.

### Detection of HPV L1 and E6-E7 genes in clinical samples with ctPCR

**Figure 5:**
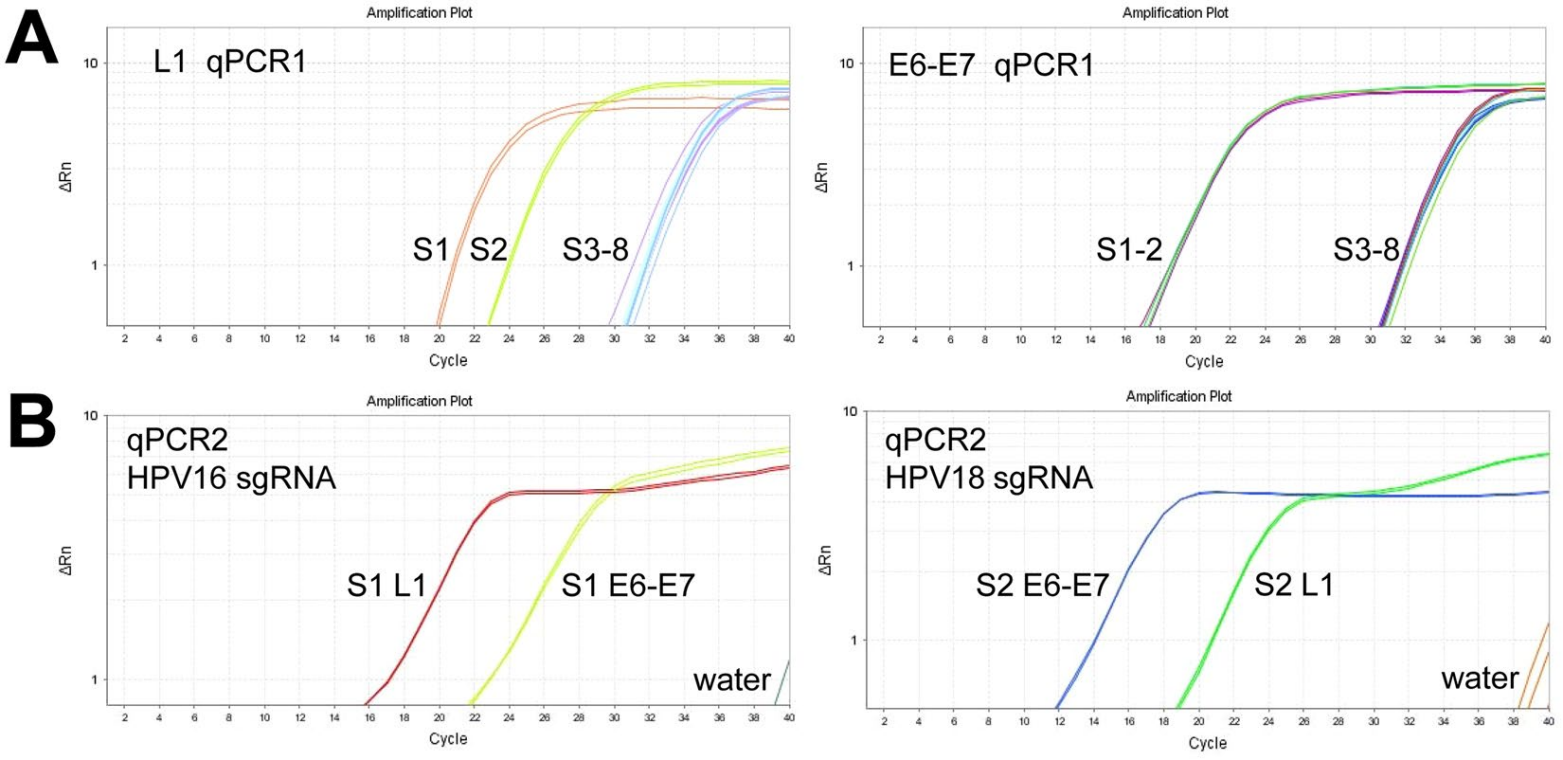
Detection of HPV in eight clinical samples with qPCR-based ctPCR. (**A**) Amplification of HPV L1 and E6-E7gene in clinical samples with qPCR1. (**B**) Detection of HPV16 and HPV18 L1 and E6-E7 genes in the HPV-positive clinical samples with qPCR2. S, sample. QPCR1 used the universal primers, L1-MY09/11 and E67-6F/7 R (Table 2). QPCR2 used the gs-primers (Table 2).

Finally, we detected the HPV L1 and E6-E7 genes in eight clinical samples (cervical mucus exfoliated cells) with ctPCR. The results revealed that the HPVs were found in two clinical samples (number 1, 2) by qPCR1 using universal primers MY09/11 and E6-6F/7R (Fig. 5A). The followed qPCR2 detection indicated that one HPV-positive clinical sample was infected by HPV16 (number 1), and the other HPV-positive clinical sample (number 2) was infected by HPV18 (Fig. 5B). These results agree with the HC2 (Diogenes) test reports of HPVs that were from Jinling Hospital, Nanjing University School of Medicine, suggesting the reliability of ctPCR detection.

**Figure 6:**
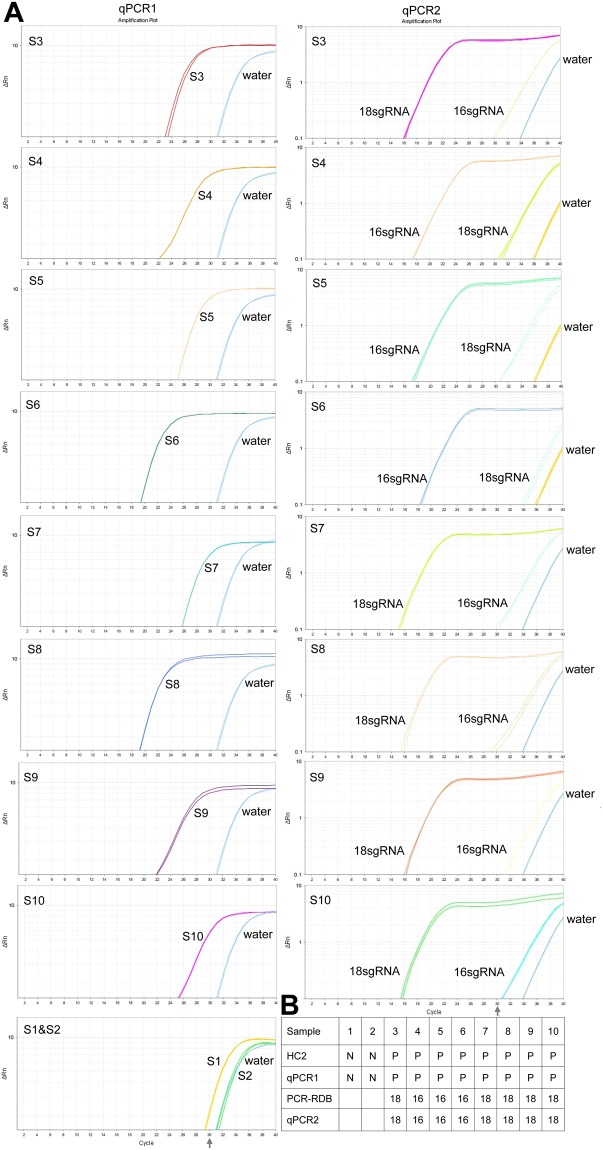
Detection of HPVs in ten clinical samples with qPCR-based ctPCR. (**A**) Detection of HPV L1 gene in clinical samples with qPCR1 and detection of HPV16 and HPV18 L1 genes in the HPV-positive clinical samples with qPCR2. (**B**) Comparison of the results of ctPCR and HC2-RDB detections. S, sample. QPCR1 used the universal primers, L1-MY09/11 (Table 2). QPCR2 used the gs-primers (Table 2). RDB, reverse dot blot. N, negative; P, positive; 16, HPV16; 18, HPV18.

To further demonstrate the capability and specificity of our method to detect clinical samples, we performed a double-blind detection of HPV clinical samples with ctPCR. We got 10 new HPV clinical samples from Jinling Hospital. These samples were firstly detected with the commercialized HPV detection kits, in which the HC2 method was used to determine if a sample is infected by high-risk HPVS and the PCR-RDB method was used to determine genotypes. These samples were then numbered and blindly detected again by our lab with ctPCR. When the detections were finished, we compared our detection results with those obtained by Jinling Hospital. The results showed that our method accurately detected all samples (Fig. 6).

### Detection sensitivity of qPCR-based ctPCR

**Figure 7:**
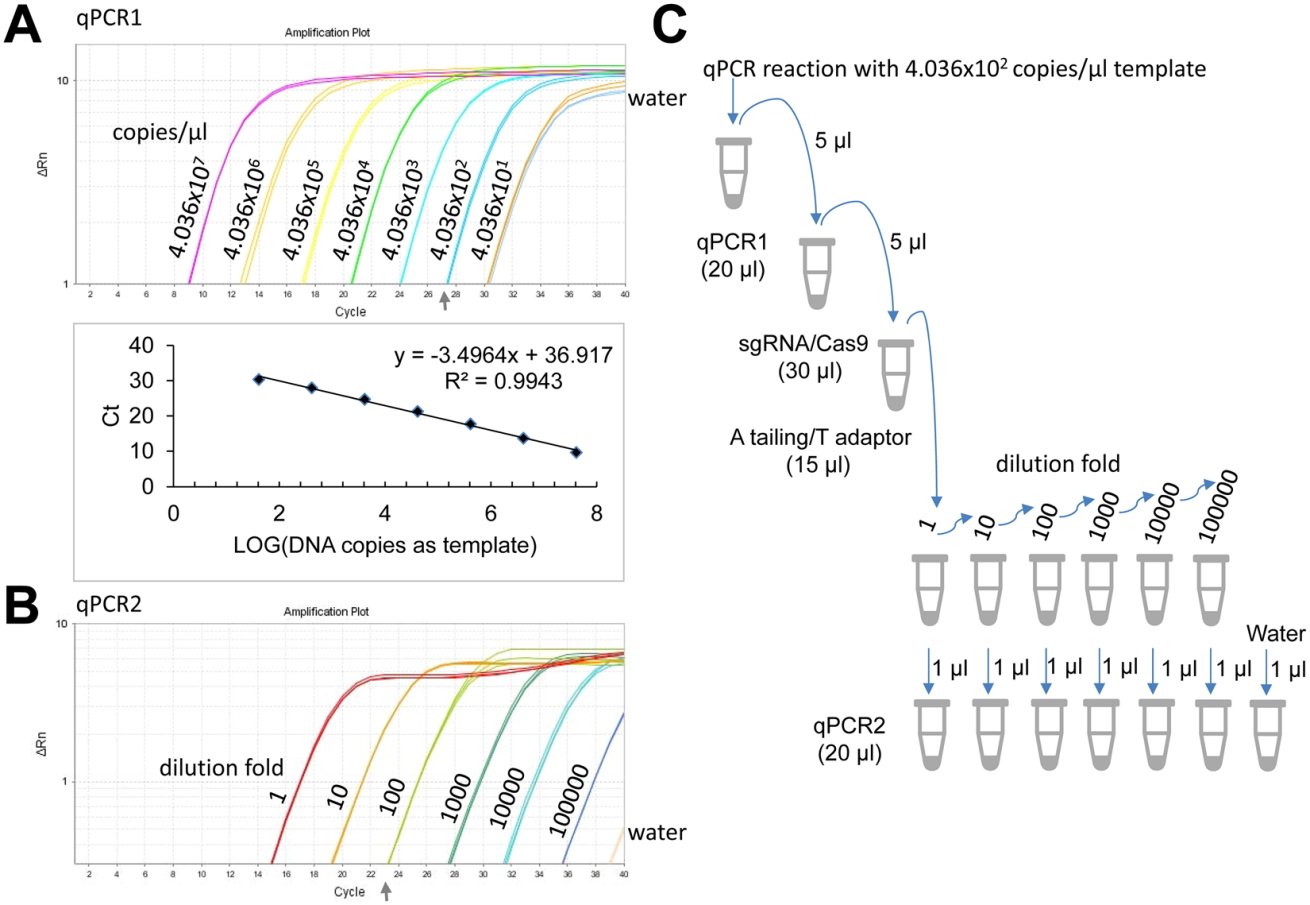
Characterization of detection sensitivity of qPCR-based ctPCR. (**A**) Detection of a HPV18 L1 gene fragment (452 bp) in various dilutions with qPCR1 by using MY09/11 primers. (**B**) Detection of the CAT-treated qPCR1 products with qPCR2 by using gs-primers (gs-18L1-F and gs-18L1-R). (**C**) Schematic show of ctPCR detection operation.

The detection sensitivity of ctPCR detection was further investigated with a standard DNA fragment. For this end, we prepared a 452-bp long HPV18 L1 DNA fragment by PCR amplification. We detected various dilutions of this DNA fragment with qPCR1 by using the MY09 and MY11 primers. Based on the Ct values and DNA copies, we built a standard curve with R^2^ over 0.99, which revealed that DNA molecules at the concentration of 400 copies/μl could be detected by qPCR1 (Fig. 7). We then detected the various dilutions of CAT-treated qPCR1 product (400 copies/μl template) with qPCR2. The results revealed that the dilution of 1000 fold could also be detected by qPCR2 (Fig. 7). This experiment demonstrated that both qPCR1 and qPCR2 had high sensitivity.

## Discussion

HPV is a kind of dsDNA virus that is the pathogen of cervical, anogenital, and other cancers^20–22^. There are around 100 HPV subtypes with different genetic variations^23^. According to the oncogenic potential, HPVs are divided into high- and low-risk HPVs^24^. The most common world-wide high-risk HPVs are HPV16 and HPV18^25–27^, which leads to approximately 70% of cervical cancers^28^. Other high-risk HPVs include HPV31, 33, 35, 39, 45, 51, 52, 56, 58, 59, 66, and 68^29^. The low-risk HPVs include HPV6, 11, 40, 42, 43, 44, 61, and 81^30,31^. Due to its carcinogenesis, at present, HPV detection is widely performed in cervical cancer diagnosis and routine women health examination mainly by using various PCR-based techniques^27,28^. For example, the Roche cobas HPV testing (cobas 4800 or cobas 6800/8800 Systems) is clinically validated for HPV primary screening. The cobas HPV assays provide specific genotyping information for HPV16 and HPV18, while simultaneously reporting the 12 other high-risk HPV types as a pooled result, all in one test and from one patient sample. Because of highly variable genotypes, HPV is an ideal material for testifying the nucleic acid detection and genotyping methods.

The L1 gene has been widely used to detect and type HPVs. In this study, we firstly designed sgRNAs for detecting HPV L1 genes because we have a set of plasmids cloned with L1 genes of various HPV subtypes (Fig. S3). We performed the *in vivo* and *in vitro* cutting assays of HPV L1 genes with Cas9-sgRNA. We also verified the ctPCR method by detecting the HPV16 and HPV18 L1 genes, which helped us to improve the ctPCR method by introducing gs-primers. We also finally detected two high-risk HPVs, HPV16 and HPV18, in two cervical carcinoma cells, HeLa and SiHa, by detecting the L1 genes with ctPCR. Additionally, it was found that HPV18 integrated into the human genome in almost all HPV18-positive cervical cancers, and HPV16 integrated into the human genome in about 60% of HPV16-positive cervical cancers^22^. However, it has been reported that HPV can lose its partial L1 gene when integrating into host cell genome, which can thus lead to false-negative detection^22,32,33^. Therefore, HPV detection is increasingly relying on the carcinogenesis gene E6/E7^34^, which can avoid missed detection because E6/E7 always exists after integration. Therefore, the E6/E7 genes can be used as the reliable targets of HPV detection. This study thus also detected E6/E7 genes when detecting HPV16 and HPV18 in three human cervical cancer cell lines, HeLa, SiHa and C-33a. The results demonstrated that the two highest-risk HPVs, HPV16 and HPV18, could be detected from SiHa and HeLa cells, respectively. However, the both HPVs were not found in C-33a. This is in agreement with the facts that HeLa is a HPV18-positive cell^17^, SiHa is a HPV16-positive cell^18^, but C-33a is a HPV-negative cell^19^.

The partial L1 missing in the integrated HPV DNA was also observed by this study. In this study, we initially designed a pair of sgRNAs for both HPV16 and HPV18 L1 genes (16–1274/16–950; 18–1490/18–1274; Fig. 1). We performed the *in vivo* and *in vitro* cutting assays of L1 plasmid using these sgRNAs (Figs 1 and 2 and S2 and S3). However, when detected HPV18 in HeLa cells using sgRNA 18–1490/18–1274, we found that no DNA could be amplified by ctPCR. Because it was reported that the L1 gene can be detected in the HeLa cells by using universal primers, MY09/11^35^, we thus designed a new pair of sgRNAs for HPV16 and HPV18 L1 genes (L1-1 and L1-2 for HPV16; L1-5 and L1-11 for HPV18; Table 1 and Fig. 3), which locate in the L1 region that can be amplified by primer MY09/11. We re-amplified L1 gene in three cervical carcinoma cell lines. As a result, the HPV L1 gene was found in the HeLa and SiHa. We then typed HPV L1 gene of two cells with ctPCR, which demonstrated that HPV16 and 18 L1 genes were present in SiHa and HeLa cells, respectively. These data suggest that the L1 region that was targeted by the initially designed sgRNAs was missed in the integrated HPV DNA. Nevertheless, the *in vivo* cutting and *in vitro* ctPCR detection of HPVs with two pairs of sgRNAs suggest that the multiple subtype-specific sgRNA can be easily designed due to the wide presence of PAMs in genome and high targeting specificity of Cas9-sgRNA system. It means that ctPCR relying on sgRNAs has higher genotyping capability than the traditional PCR that depends on the specific primers.

In this study, when detecting HPV16 and HPV18 DNAs in cervical carcinoma cell lines, we used a two-round PCR strategy. The first round PCR amplified L1 gene with universal primer MY09/11 or E6-E7 genes with universal primer E67-6F/7R. The PCR products were treated with CAT. Then the second round of PCR was performed with a general primer or a pair of general-specific primers (gs-primers). Therefore, the first-round PCR was used to amplify the HPV DNA for judging whether a sample was infected by HPVs. The second-round PCR was used to type or discriminate the virus infected the sample. Due to the high sensitivity of PCR amplification, the detection limit was warranted by the first round PCR (PCR1). Additionally, PCR1 also provided enough target DNA for the followed ctPCR.

DNA typing is critical for the specific DNA detection, which is especially useful for the discriminative detection of virus subtypes and polymorphic DNAs. In this study, to guarantee the specificity of ctPCR detection, we adopted two strategies. One is to design two highly specific sgRNA commonly targeting to the detected DNA type. The other is to use a pair of gs-primers in ctPCR. Although the off-target of Cas9-sgRNA system is now limiting its application in human gene therapy, this disadvantage did not affect the ctPCR detection due to the double sgRNA cutting in a short DNA region. Additionally, the distant off-targets beyond the PCR amplification limit also did not affect the ctPCR. Even the off-targets cutting produced a fragment that is in the range of PCR amplification, these off-targets can be prevented from ctPCR amplification by the gs-primers. The gs-primers further guarantee the detection specificity of ctPCR on the base of a pair of specific sgRNAs. Therefore, this study demonstrated that ctPCR could specifically detect the L1 and E6-E7 genes of two highest risk HPVs (HPV16 and HPV18) integrated in the complicated genomic DNAs of two human cervical carcinoma cell lines, HeLa and SiHa. This study also demonstrated that ctPCR could specifically detect the L1 genes of the two highest risk HPVs infected in clinical sample. These data indicate the high specificity of ctPCR.

Real-time PCR has already become a widely popularized DNA detection tool in clinics. For the clinical application, this study investigated the feasibility to realize ctPCR detection with qPCR. It was found that the Cas9-sgRNA cutting could be directly performed in the qPCR1 solution, indicating that the reagents of qPCR1 reaction did not interfere the followed Cas9-sgRNA cutting. Similarly, the reagents of Cas9-sgRNA reaction did not interfere the subsequent A tailing and T adaptor ligation, which also did not interfere the final qPCR2. Therefore, the whole ctPCR detection process was free of DNA purification and just realized by simply transferring solutions of three-steps (Fig. 4A). This kind of compatibility among various biochemical reactions of different functions made the ctPCR detection realizable in clinical detection easily and rapidly. In addition, the qPCR-based ctPCR also increased the detection limit.

Clearly, the test cycle of ctPCR detection is mainly dependent on two round of PCR. In this study, we have in fact optimized the CAT steps. We thus provided an optimized CAT treatment program of shortest time (Figs 3 and 4). We found that five minutes was enough for Cas9-sgRNA to cut its targets in the ctPCR detection, indicating the high *in vitro* cutting efficiency of Cas9-sgRNA^36^. We also found that five minutes were enough for A tailing and T adaptor ligation, respectively. Therefore, the whole CAT treatment process can be finished as few as 15 min. In fact, PCR1 contributes to the high efficiency of Cas9-sgRNA cutting due to the enrichment of targets. In this study, we have ever tried detecting HPV18 in the HeLa gDNA as we detected L1 plasmid; however, we failed, even after cutting large amount of gDNA (1 μg) for a long time (2 h). This agrees with a recent finding that Cas9-sgRNA complex spends as long as 6 hours to find its target in the *E*. *coli* genome (about 4 million base pairs)^37^. Therefore, it will take a long time to find low-copy targets in a highly complicated DNA context such as human gDNA. This case can be further deteriorated by the fact that Cas9 is a single-turnover enzyme in DNA cutting^36^. This problem may be resulted from low copies of HPV18 integrated in the HeLa gDNA, which may be addressed by using more Cas9-sgRNA complex and long-time digestion. However, a time-consuming and costly detection is prohibitive to clinical application. Nevertheless, a PCR1-free ctPCR should be effective to high-copy targets as we detected the L1 plasmid.

It should be noted that this study focused on validating the ctPCR method in proof-of-concept by using HPV as a convenient available experimental material. The method could also be used to detect other interested DNA. This study employs HPV DNA as DNA target for ctPCR detection. The results indicate that ctPCR can detect and type HPV DNAs. It was found that ctPCR could detect the HPV L1 gene in as little as 5 pg gDNA from cervical carcinoma cell. It was also found that ctPCR could detect and type HPVs in clinical samples.

## Conclusion

This study developed a new method for detecting target DNA based on Cas9 nuclease, which was named as ctPCR, representing the Cas9-sgRNA-typing PCR. This method was verified by detecting the L1 genes of two high-risk HPVs (HPV16 and HPV18) from 11 HPV subtypes successfully. This method was also verified by successfully detecting the L1 and E6/E7 genes of two high-risk HPVs (HPV16 and HPV18) in three cervical carcinoma cells (HeLa, SiHa and C-33a) and the L1 and E6-E7 genes of HPVs in clinical samples. This study demonstrated that ctPCR has high specificity and sensitivity. This study also demonstrated that ctPCR detection could be realized by a simple two-round qPCR, making ctPCR applicable to clinical diagnosis. By using the widely available qPCR machines, the whole ctPCR detection process can be finished in as few as 3 to 4 hours. Therefore, ctPCR should be useful in DNA detection and genotyping in the future.

## Electronic supplementary material

Supplementary Information
