# Supplementary material for: CRISPR-typing PCR (ctPCR), a new Cas9-based DNA detection method
Source: Sci Rep. 2018 Sep 20;8:14126. doi: 10.1038/s41598-018-32329-x (PMC6148268; doi:10.1038/s41598-018-32329-x)
Supplement: Supplementary file 1 — Supplementary Information [file 41598_2018_32329_MOESM1_ESM.pdf]

Supplementary Information

CRISPR-typing PCR (ctPCR), a new Cas9-based DNA detection method

Qiao Wang[1], Beibei Zhang[1], Xinhui Xu[1], Feifei Long[2], Jinke Wang[1]*

[1] *State Key Laboratory of Bioelectronics, Southeast University, Nanjing 210096, China*
[2] *Nanjing Foreign Language School, Nanjing 210096, China*

*Correspondence should be addressed to Jinke Wang:
Tel.: +86 25 83793620; fax: +86 25 83793620.
E-mail address: wangjinke@seu.edu.cn

Materials and methods

Construction of sgRNA expression plasmid

A pair of primers were firstly synthesized to amplify the prokaryotic Cas9 gene sequence with PCR from pCas9 (Addgene), in which the forward primer contained the J23100 promoter plus RBS sequences (total 88 bp). Then the prokaryotic Cas9 gene sequence was amplified from pCas9 (Addgene) by using the synthesized primers. The PCR product was cloned the whole Cas9, trancRNA and spacer RNA sequences from a pCas9 plasmid removed. The J23119-sgRNA sequence was amplified with PCR from pgRNA-bacteria (Addgene) and cloned into the newly prepared pCas9 vector. The new plasmid that can express Cas9 protein and sgRNA, which was named as pCas9-sgRNA. The Cas9 protein was under the control of J23100 promoter and the sgRNA was under the control of J23119 promoter. This plasmid also contained a chloromycetin gene under control of cat promoter. The various sgRNA sequences (annealed double-stranded oligonucleotides ended with BsaI sites) were then cloned into pCas9-sgRNA by using BsaI for simultaneously expressing Cas9 protein and the interested sgRNA in bacteria. The target sequences of sgRNA (plus PAM) used for the in vivo Cas9/sgRNA cutting were shown in Table 1.

Preparation of sgRNA

The sgRNA was synthesized by an *in vitro* transcription using T7 polymerase (New England Biolabs) according to the manufacturer's instruction. In brief, sgRNA templates were generated by a three-round fusion PCR amplification using oligonucleotides listed in Table 2. The 1st-PCR was amplified with F1 and R (7 cycles); the 2nd-PCR was amplified with primers F2 and sgR using the 1st-PCR product as template (30 cycles), and the 3rd-PCR was amplified with primers F3 and sgR using the 2nd-PCR product as template (30 cycles). The purified 3rd-PCR products were used as template for preparing sgRNA by *in vitro* transcription. The in vitro transcription was performed by incubating the purified sgRNA template with T7 RNA polymerase (New England Biolabs) supplemented with rNTPs (New England Biolabs) overnight at 37 °C. The in vitro transcribed RNA was mixed with Trizol solution, and then successively extracted with chloroform and isopropanol, and precipitated with ethanol. Purified RNA was dissolved in the RNase-free ddH_2O and quantified by spectrometry. A detailed protocol for preparing sgRNA was as follows. The sequences of sgRNAs and their target HPV subtypes and genes are shown in Table 1.

Preparation of sgRNA in vitro transcription template

PCR1: According to the skeletal portion of the sgRNA, a pair of primers (F1 and R as shown in Table S1) was first designed for PCR. PCR reaction system (30 μL): 2 μL F1 (Table S1), 2 μL R (Table S1), 15 μL 2×primestar (TAKARA), and the volume was supplemented to 30 μL with H_2O. PCR reaction program: 95°C for 2 min; 7 cycles: 95°C for 15 s, 72°C for 1 min. Then the PCR product was successively electrophoresed on a 1.5% agarose gel at 100 V for 40 min, recovered by a gel recovery kit (Axygen), and dissolved in 25 μL of the eluate. The DNA concentration and purity were measured by a Nanodrop 2000 spectrophotometer. The PCR product was named as Fragment 1 and kept at -20 °C for future use.

PCR2: PCR was performed using Fragment 1 as a template and F2 and Sg-R as primers. PCR reaction system (50 μL): 2 μL Fragment 1, 1 μL F2, 1 μL Sg-R, 25 μL 2×primestar (TAKARA), 20 μL H_2O. PCR reaction program: 95°C for 2 min; 30 cycles: 95°C for 15 s, 60°C for 30 s, 72°C for 1 min; 72°C for 2 min. At the end of the PCR, the PCR product was directly recovered using a PCR cleaning kit (Axygen) and dissolved in 25 μL of the eluate. The DNA concentration and purity of the PCR product were measured with a Nanodrop 2000 spectrophotometer. The PCR product was named as Fragment 2 and kept at -20 °C for future use.

PCR3: PCR was performed using Fragment 2 as a template, F3 (Table S1) and Sg-R (Table S1) as primers.

PCR reaction system (50 μl): 2 μL Fragment 2, 1 μL F3 (Table S1), 1 μL Sg-R (Table S1), 25 μL 2× primestar (TAKARA), 20 μL H_2O. PCR reaction program: 95°C for 2 min; 30 cycles: 95°C for 15 s, 60°C for 30 s, 72°C for 1 min; 72°C for 2 min. The PCR product was directly recovered with a PCR cleaning kit (Axygen) and dissolved in 25 μL of the eluate. The DNA concentration and purity of the PCR product were measured with a Nanodrop 2000 spectrophotometer. This PCR product was designated as T7-sgRNA transcription template and kept at -20 °C for future use.

Preparation of sgRNA by in vitro transcription

The in vitro transcription was performed according to the reference system and dosage in the purchased T7 RNA Pol (NEB) specification. The H_2O, EP tube, and pipette tips used in this process must all undergo RNase processing. The in vitro transcription reaction (20 μL) includes: 0.2~1 μg T7-sgRNA transcription template, 2 μL T7 RNA Pol, 2 μL T7 RNA Pol buffer, 1 μL rNTP (NEB), supplemented with H_2O to 20 μL. The reaction was incubated at 37°C overnight.

Extraction and purification of sgRNA

RNA was extracted using Trizol (Invitrogen) reagents. First, 1 mL Trizol was added to the overnight-reacted in vitro transcription reaction and pipette several times. The lysate was transferred to a 1.5 mL centrifuge tube and allowed to stand at room temperature for 5 min. Chloroform was added in an amount of 0.2 ml chloroform/mL Trizol. The tube was capped, vigorously shaken for 15 s, allowed to stand at room temperature for 5 min, and centrifuged at 12000 g for 15 min at 4°C. The upper liquid phase was transferred to a clean centrifuge tube and added isopropanol (0.5 mL/mL Trizol). The mixture was mixed gently by inverting several times, allowed to stand at room temperature for 10 min and centrifuged at 12,000 g for 10 min at 4°C. The supernatant was discarded. The pellet was added 75% ethanol (1 mL/mL Trizol), mixed well and centrifuged at 7500 g for 5 min at 4°C. The supernatant was removed and the pellet was incubated at room temperature for 5 to 10 min to allow it to dry naturally (do not dry completely). The pellet was added 30 μL of DEPC to dissolve the RNA. The purity and concentration of RNA was determined using a UV spectrophotometer. One μg was subjected to 1.5% agarose gel electrophoresis.

Table S1. Oligonucleotides used to prepare the transcriptional template of sgRNA in this study

Name	Primers (5' to 3') (italic bases are sgRNA sequence)
F1	GTTTTAGAGCTAGAAATAGCAAGTTAAAATAAGGCTAGTCCGTTATCAACTTG
R	AAAAAAAAGCACCGACTCGGTGCCACTTTTTCAAGTTGATAACGGACTAGCC
sgR	AAAAAAAAGCACCGACTCGGTGCCACTTTTTC
16-950-F2	*ACTGTGTTTATTAACTCTAAG*TTTTAGAGCTAGAAATAGCAAG
16-950-F3	TTCTAATACGACTCACTATAG*ACTGTGTTTATTAACTCTAAG*
16-1274-F2	*AAACCAAACTTATTGGGGTC*GTTTTAGAGCTAGAAATAGCAAG
16-1274-F3	TTCTAATACGACTCACTATAG*AAACCAAACTTATTGGGGTC*
18-1274-F2	*AAACCAAATTTATTTGGGTC*GTTTTAGAGCTAGAAATAGCAAG
18-1274-F3	TTCTAATACGACTCACTATAG*AAACCAAATTTATTTGGGTC*
18-1490-F2	*AGATATACGGTATTGTCACTG*TTTTAGAGCTAGAAATAGCAAG
18-1490-F3	TTCTAATACGACTCACTATAG*AGATATACGGTATTGTCACTG*
16E6-7-F2	*GATTCCATAATATAAGGGGT*GTTTTAGAGCTAGAAATAGCAAG
16E6-7-F3	TTCTAATACGACTCACTATA**G***GATTCCATAATATAAGGGGT*
16E7-6-F2	*GAGGAGGAGGATGAAATAGA*GTTTTAGAGCTAGAAATAGCAAG
16E7-6-F3	TTCTAATACGACTCACTATA**G***GAGGAGGAGGATGAAATAGA*
16L1-1-F2	*GGAGTACCTACGACATGGGG*GTTTTAGAGCTAGAAATAGCAAG
16L1-1-F3	TTCTAATACGACTCACTATA**G***GGAGTACCTACGACATGGGG*
16L1-2-F2	*GGATCTTCTTTAGGTGCTGG*GTTTTAGAGCTAGAAATAGCAAG
16L1-2-F3	TTCTAATACGACTCACTATA**G***GGATCTTCTTTAGGTGCTGG*
18E6-10-F	*GTGCTGCAACCGAGCACGAC*GTTTTAGAGCTAGAAATAGCAAG
18E6-10-F	TTCTAATACGACTCACTATA**G***GTGCTGCAACCGAGCACGAC*
18E7-1-F2	*CGAGCAATTAAGCGACTCAG*GTTTTAGAGCTAGAAATAGCAAG
18E7-1-F3	TTCTAATACGACTCACTATA**G***CGAGCAATTAAGCGACTCAG*
18L1-5-F2	*GCATCATATTGCCCAGGTAC*GTTTTAGAGCTAGAAATAGCAAG
18L1-5-F3	TTCTAATACGACTCACTATA**G***GCATCATATTGCCCAGGTAC*
18L1-11-F2	*TGTTGCTATTACCTGTCAAA*GTTTTAGAGCTAGAAATAGCAAG
18L1-11-F3	TTCTAATACGACTCACTATA**G***TGTTGCTATTACCTGTCAAA*

Figures:

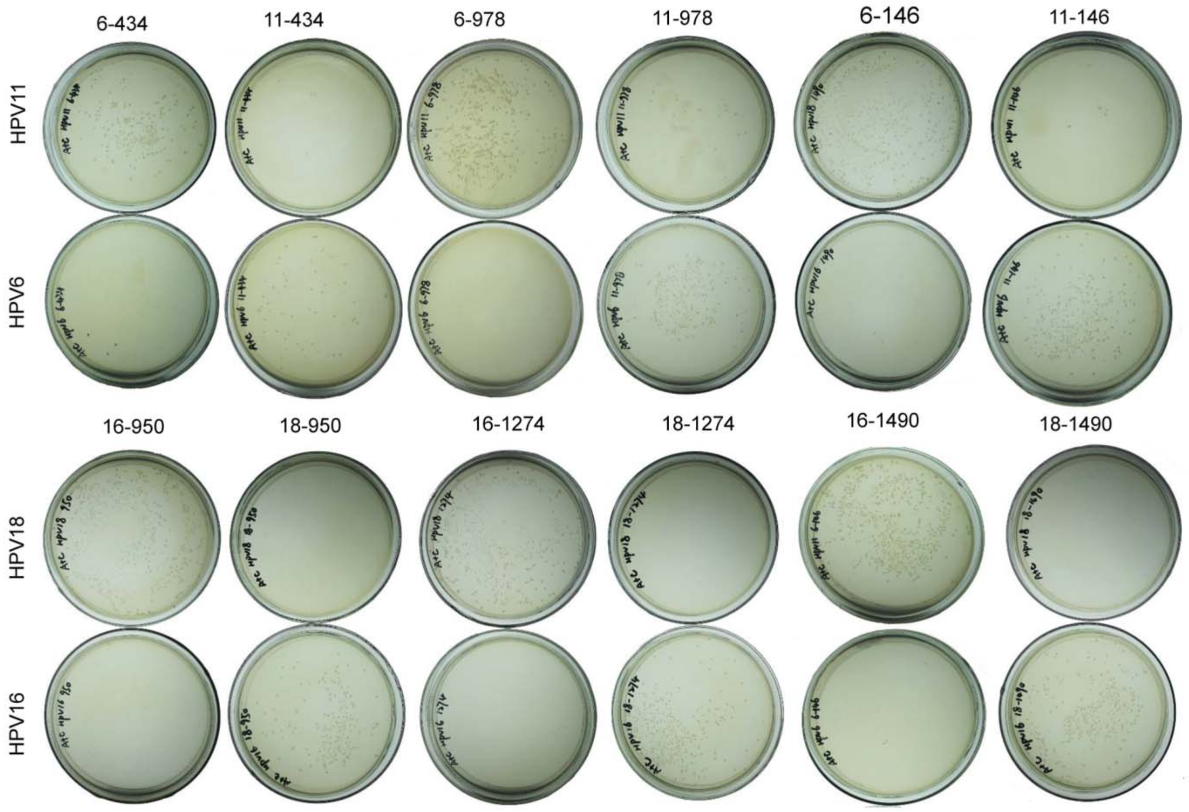

Fig.S1 *In vivo* cutting of HPV L1 genes with Cas9/sgRNA. The *E.coli* DH5α was firstly transformed with HPV L1 plasmid and the selected Amp-resistant cells were then transformed with Cas9/sgRNA plasmid. The transformed cells were cultivated on agars with ampicillin plus chloromycetin overnight and imaged.

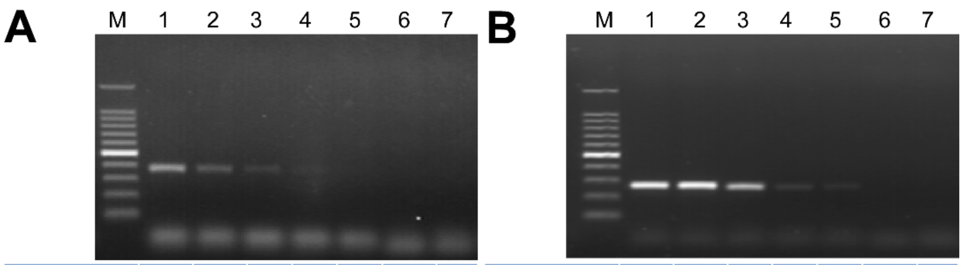

	1	2	3	4	5	6	7
pHPV16(ng)	200	100	50	10	5	200	-
AatII	+	+	+	+	+	+	-
16sgRNA/cas9	+	+	+	+	+	-	-
T adaptor	+	+	+	+	+	+	-
T4 DNA ligase	+	+	+	+	+	+	-
PCR (primers)	+	+	+	+	+	+	+

	1	2	3	4	5	6	7
pHPV18(ng)	200	100	50	10	5	200	-
AatII	+	+	+	+	+	+	-
18sgRNA/cas9	+	+	+	+	+	-	-
T adaptor	+	+	+	+	+	+	-
T4 DNA ligase	+	+	+	+	+	+	-
PCR (primers)	+	+	+	+	+	+	+

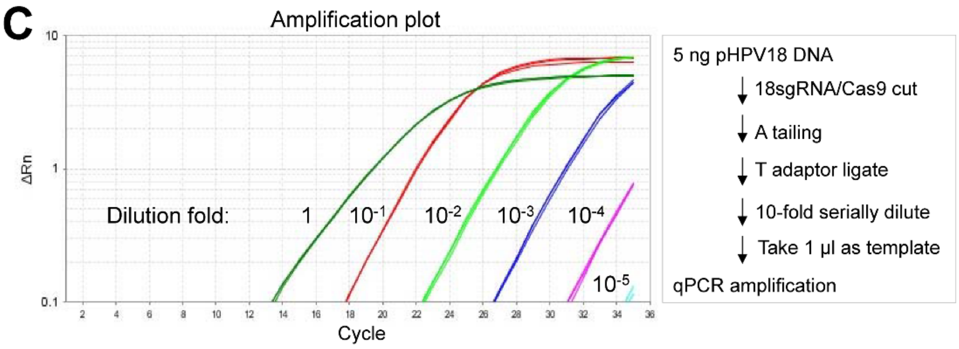

Fig.S2 Sensitivity of ctPCR detection of HPV 16 or 18 L1 genes. (A) Detection of HPV 16 L1 gene with tPCR-based ctPCR. (B) Detection of HPV 18 L1 gene with tPCR-based ctPCR. (C) Detection of HPV 18 L1 gene with qPCR-based ctPCR.

A

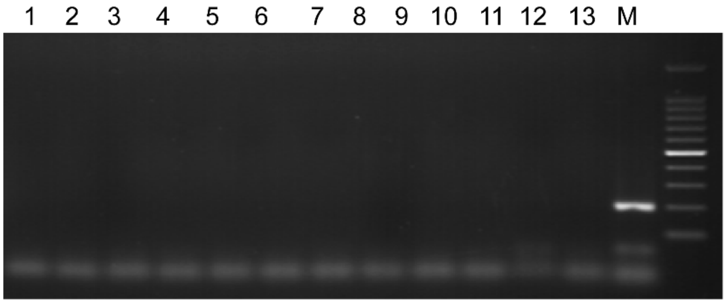

	1	2	3	4	5	6	7	8	9	10	11	12	13	M
HPV L1 DNA	66	59	58	56	52	51	45	35	33	31	18	16	16	-
16sgRNA/cas9	+	+	+	+	+	+	+	+	+	+	+	-	+	-

B

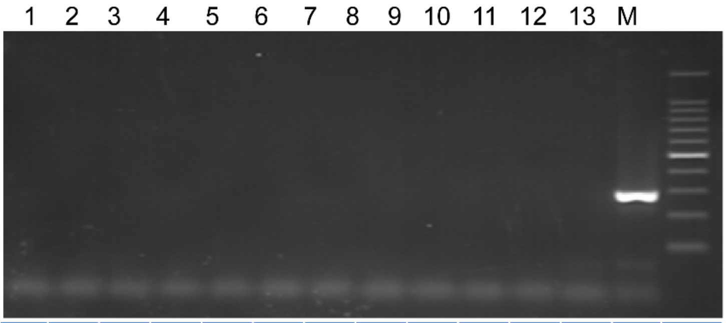

	1	2	3	4	5	6	7	8	9	10	11	12	13	M
HPV L1 DNA	66	59	58	56	52	51	45	35	33	31	16	18	18	-
18sgRNA/cas9	+	+	+	+	+	+	+	+	+	+	+	-	+	-

Fig.S3 Detection of HPV 16 and 18 L1 genes in thirteen HPV subtypes with ctPCR. (A) Detection of HPV 16 L1 gene in thirteen HPV subtypes. (B) Detection of HPV 18 L1 gene in thirteen HPV subtypes. The final ctPCR products were run with agarose gel.

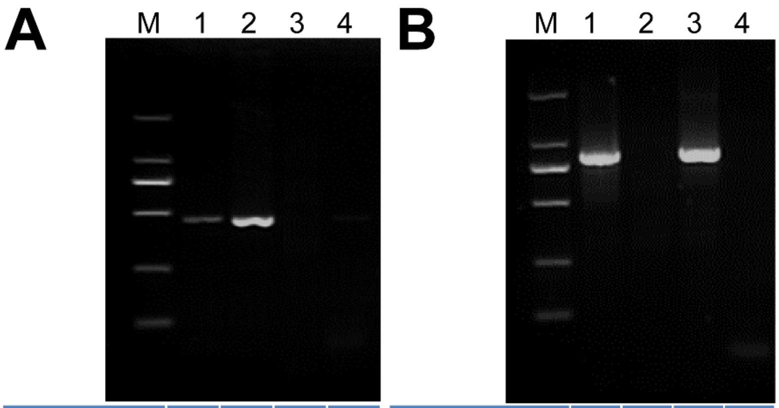

	1	2	3	4
SiHa gDNA	+	-	-	-
HeLa gDNA	-	+	-	-
C-33a gDNA	-	-	+	-
MY09/11	+	+	+	+

	1	2	3	4
SiHa gDNA	+	-	-	-
HeLa gDNA	-	-	+	-
C-33a gDNA	-	+	-	-
E67-6F/E67-7R	+	+	+	+

Fig.S4 Detection of HPV18 L1 gene in HeLa cells with qPCR-based ctPCR. (A) Detecting the HPV16/18 L1 gene in the SiHa/HeLa gDNA with qPCR1 by using universal primers MY09/11. (B) Detecting the HPV16/18 L1 gene in the SiHa/HeLa gDNA with qPCR1 by using universal primers E67-6F/7R.

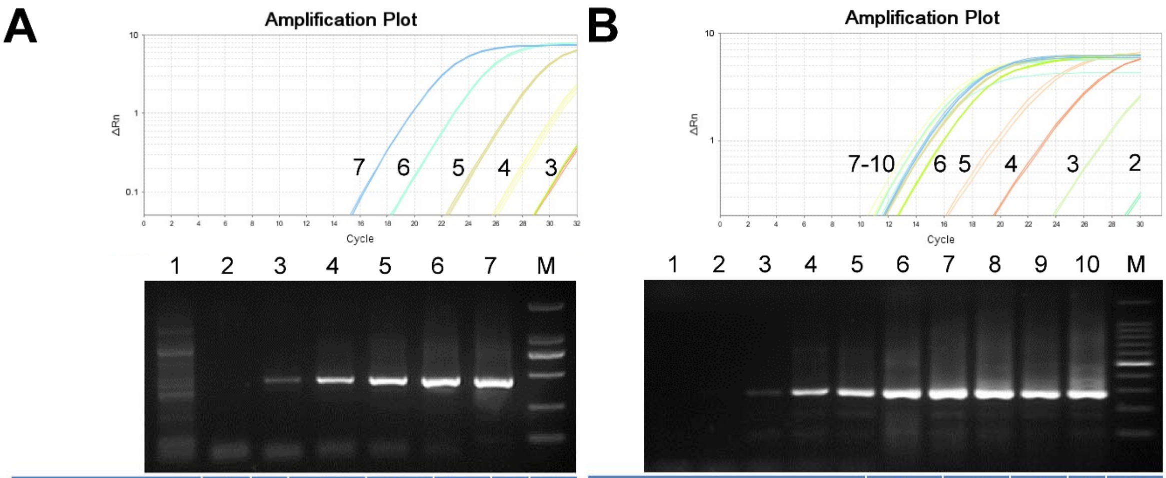

	1	2	3	4	5	6	7
HeLa gDNA (ng)	0	0	0.005	0.05	0.5	5	50
C-33a gDNA (ng)	50	0	0	0	0	0	0
qPCR1 (MY09/11)	+	+	+	+	+	+	+

	6	7	8	9	10
qPCR1 template (HeLa gDNA, ng)	0.005	0.05	0.5	5	50
18sgRNA/Cas9	+	+	+	+	+
oJW (adaptor)	+	+	+	+	+
qPCR2 template (μl)	1	1	1	1	1

	1	2	3	4	5
qPCR1 template (HeLa gDNA, ng)	0	0.005	0.005	0.005	0.005
Dilution of adapted qPCR1 products	0	10^{-4}	10^{-3}	10^{-2}	10^{-1}
PCR2 template (μl)	0	1	1	1	1

Fig.S5 Detection of HPV18 L1 gene in HeLa cells with qPCR-based ctPCR. (A) Detecting the HPV18 L1 gene in the HeLa gDNA with qPCR1 by using universal primers MY09/11. (B) Typing the HPV18 L1 gene with ctPCR. The final ctPCR products were also run with agarose gel.

Ct values of all qPCR amplification performed in this study

Fig. S2 qPCR

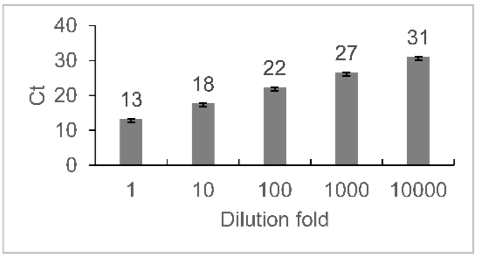

Fig.S5 qPCR

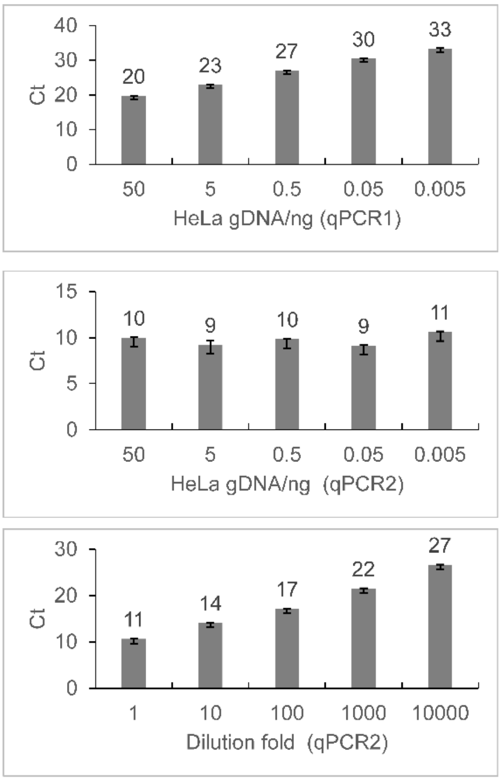

Fig.4 qPCR

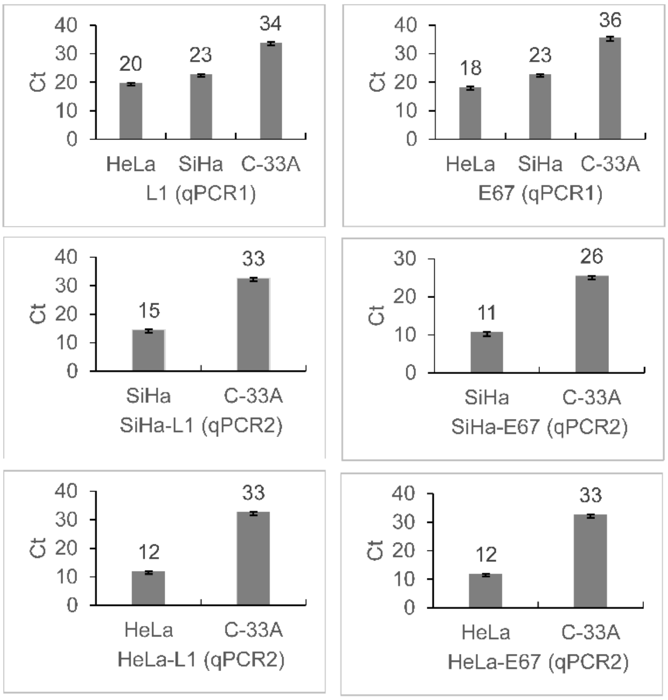

Fig.5 qPCR

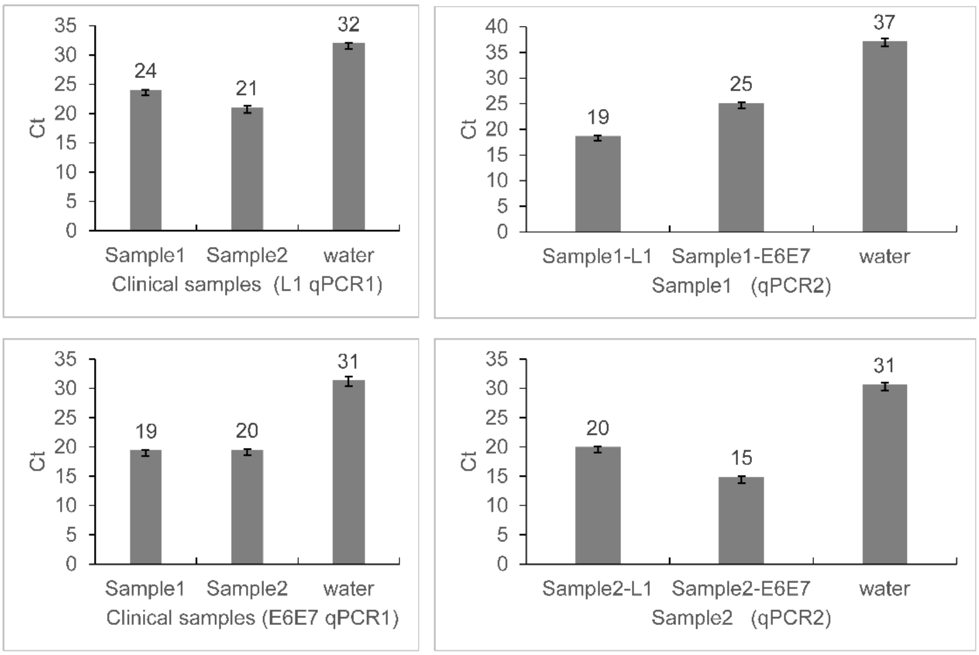

Fig.6 qPCR1

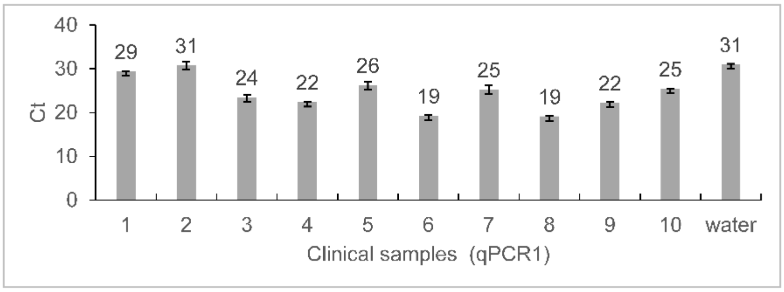

Fig.6 qPCR2

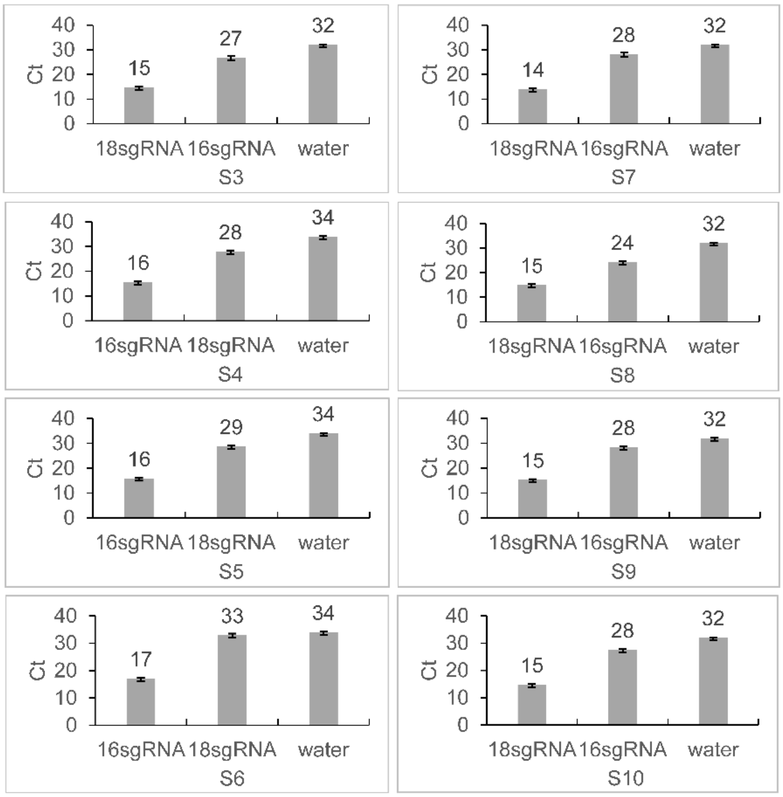

Fig.7 qPCR1

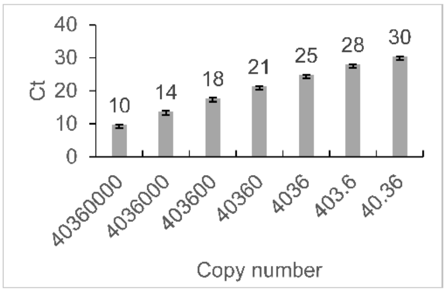

Fig.7 qPCR2

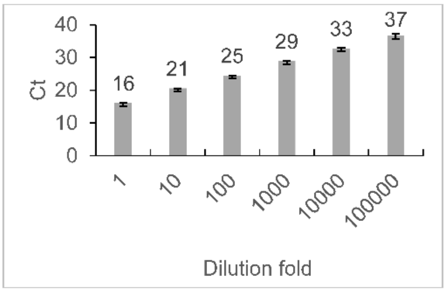

Full-length gels

(Please note: some full-length gels also contained some non-related running lanes; however, it is very clear and easy to see the used lanes in these gels.)

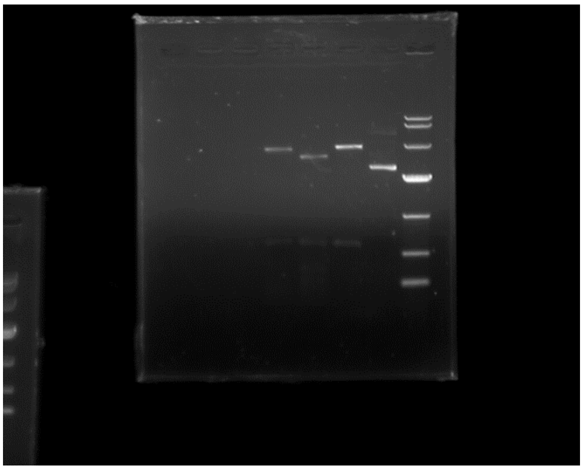

Fig. 1B

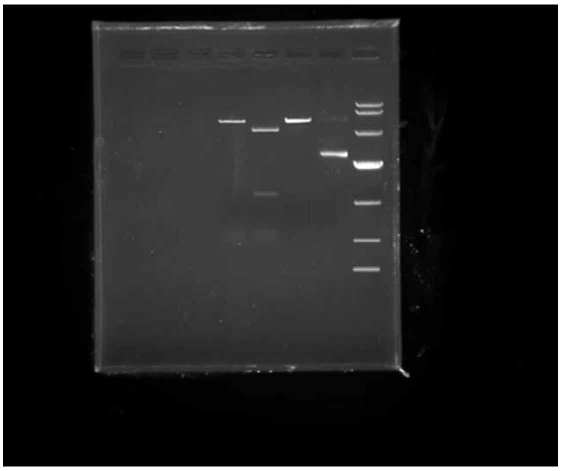

Fig. 1C

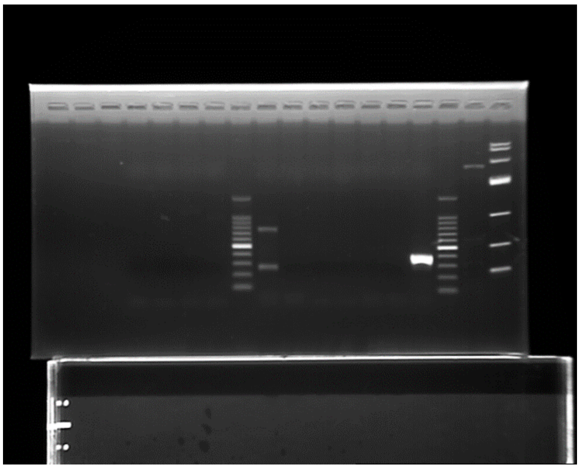

Fig. 2C-1 and 2C-2

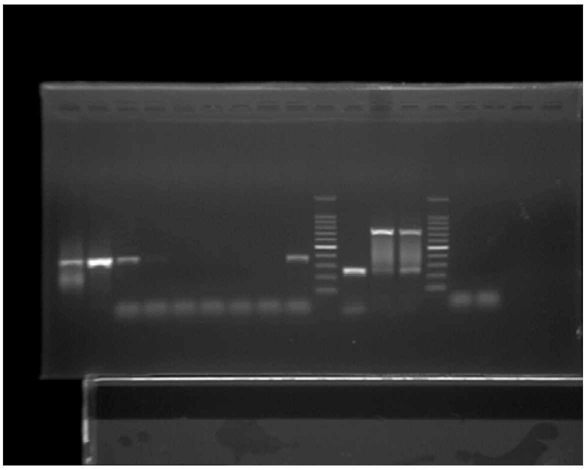

Fig.2B-1

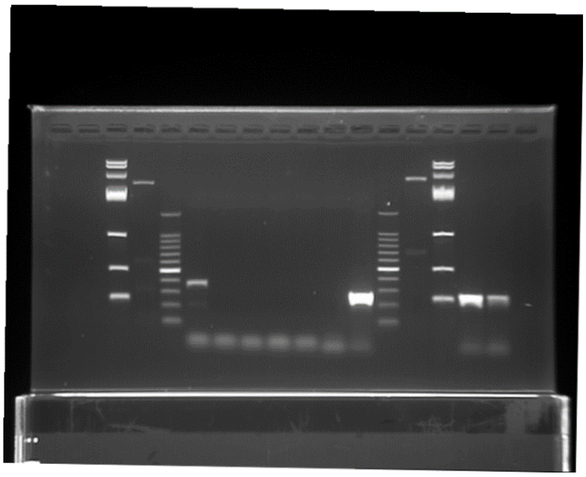

Fig. 2B-2

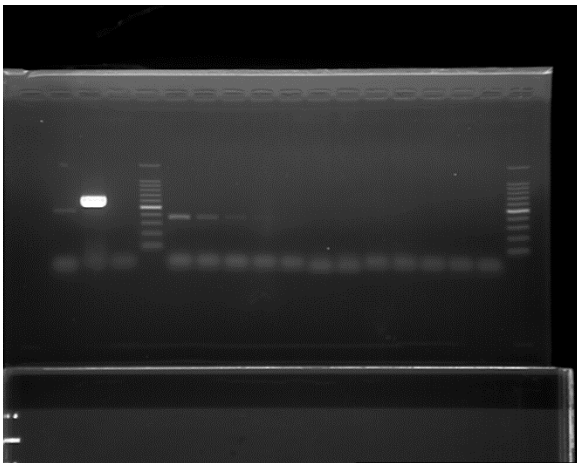

Fig. S2A

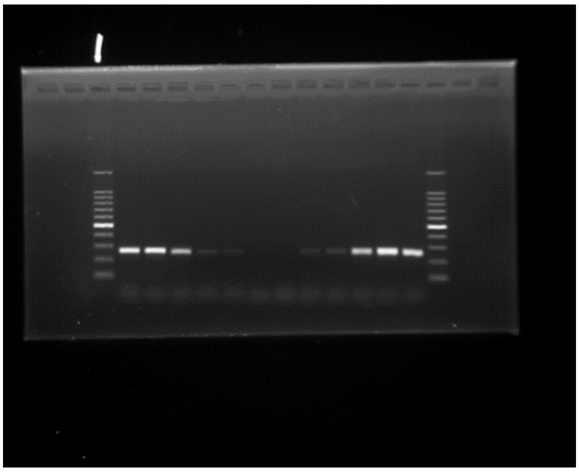

Fig. S2B

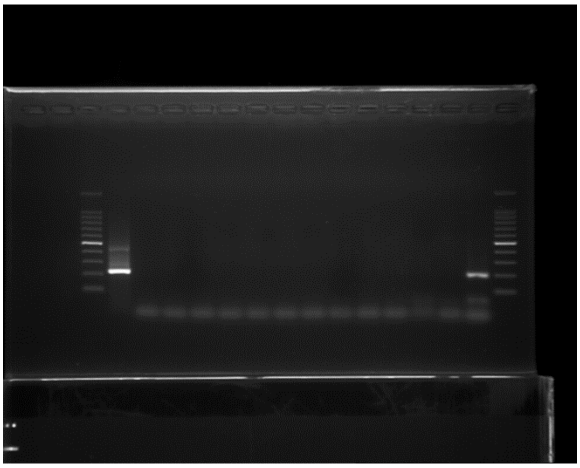

Fig. S3A

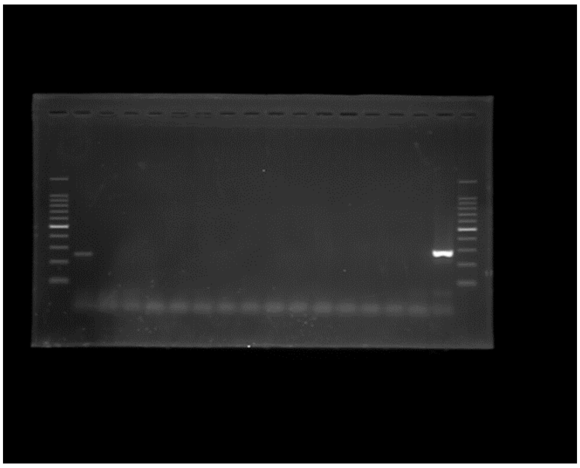

Fig. S3B

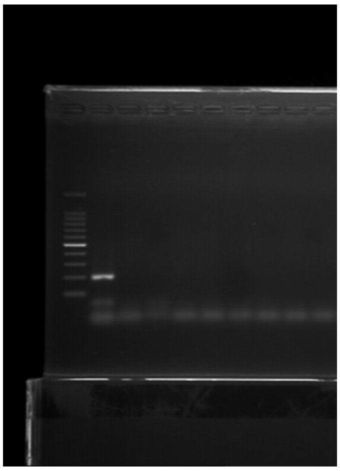

Fig. 3B-1

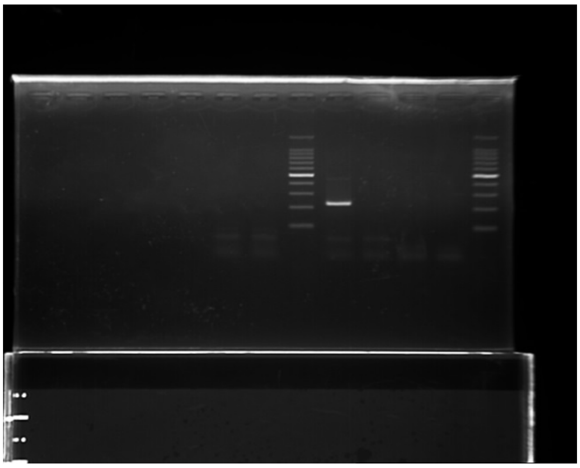

Fig. 3B-2

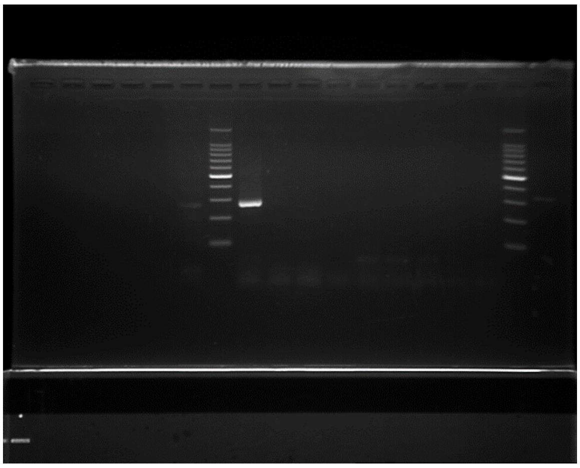

Fig. 3C-1

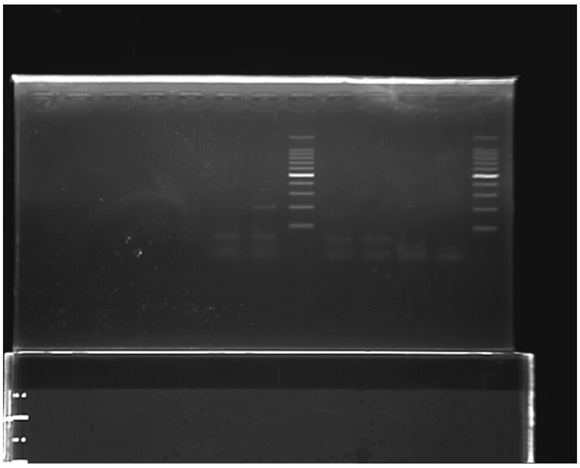

Fig. 3C-2

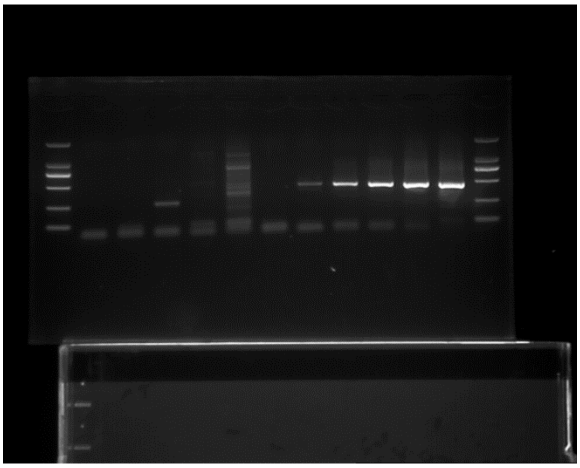

Fig.S5A

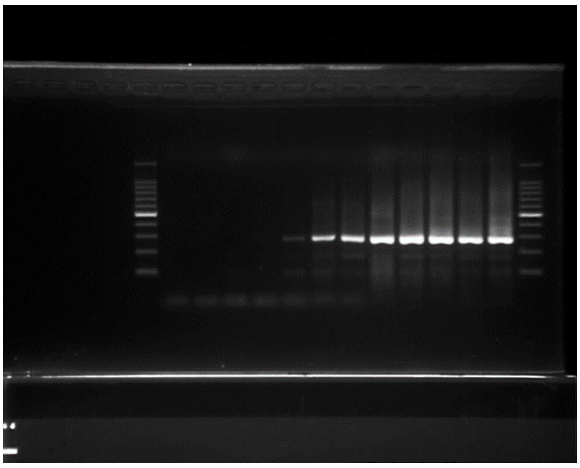

Fig. S5B
